# circGLS2 inhibits hepatocellular carcinoma recurrence via regulating hsa-miR-222-3p–PTEN–AKT signaling

**DOI:** 10.1038/s41392-022-01275-6

**Published:** 2023-02-17

**Authors:** Xi Chen, Ting Wu, Linfeng Xian, Longteng Ma, Nan Li, Wenbin Liu, Peng Cai, Xiaojie Tan, Jianhua Yin, Guangwen Cao

**Affiliations:** 1 Department of Epidemiology, Second Military Medical University, Shanghai, China; 2 Department of Surgery, Eastern Hepatobiliary Surgery Hospital, Second Military Medical University, Shanghai, China

**Keywords:** Gastrointestinal cancer, Tumour biomarkers, Molecular medicine, Non-coding RNAs, Prognostic markers

**Dear Editor**,

Growing evidences indicate that circRNAs affect hepatocellular carcinoma (HCC) progression.^1–4^ Understanding the roles of circRNAs in HCC recurrence helps develop innovated therapies. To identify novel HCC recurrence-related circRNA, thirteen HCC patients were subjected to a multi-omics analysis of mRNA, circRNA, and miRNA expression profiles by next-generation sequencing (Supplementary Fig. S1a). Of these patients, four recurred within two years after the first curative resection (primary tumors, PT) and received a second radical resection (recurrent tumors, RT), while nine had not recurred in five years after the resection (non-recurrent tumors, NRT) (Fig. 1a, Supplementary Table S1). We enrolled 110 extra HCC patients as a validation cohort (Supplementary Table S2). GSEA among the three tumor types obtained more enriched gene sets than those enriched among the paired adjacent tissues (Supplementary Fig. S1b, Table S3). In the top enriched gene sets, HCC recurrence- and tumorigenesis-related gene sets were discovered between NRT and PT or RT (Supplementary Fig. S1c). Thus, PT and RT were more malignant than NRT. Interestingly, gene sets enrichment between RT and PT suggested that recurrent HCCs were less malignant than primary counterparts (Supplementary Fig. S1c). This was also evident in the circRNA data and miRNA data (Supplementary Fig. S1d, e).

**Fig. 1:**
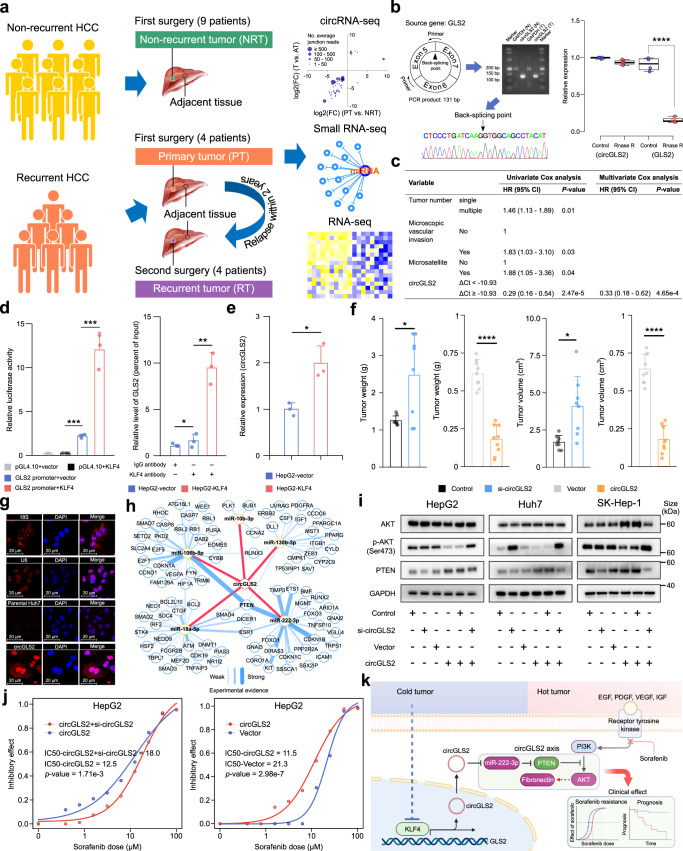
circGLS2 inhibits HCC recurrence. **a** The design of multi-omics analysis for HCC patients with and without postoperative recurrence. **b** Validation of circGLS2’s circular structure. PCR experiment and Sanger sequencing were performed sequentially. The back-splicing junction site was clearly observed. RNA templates diluted for 10 folds were applied for the examination of GAPDH (Left). After treating with RNase R, circGLS2 showed non-significant changes, while GLS2 with linear structure decreased significantly (Right). **c** The Cox regression analysis for the factors significantly affected the recurrence of postoperative HCC. **d** Luciferase reporter assay (Left) and ChIP-qPCR (Right) for KLF4 in parental and KLF4-overexpressing HepG2 cells. **e** circGLS2 was up-regulated upon ectopic express of KLF4 in HepG2 cells. **f** The weight and volume changes of tumors collected from mice injected with circGLS2 siRNA- and overexpression plasmid-transfected Huh7 cells. **g** Subcellular localization of circGLS2. circGLS2 was localized in parental and circGLS2-overexpressing Huh7 cells. 18 S and U6 served as controls localized in the cytoplasm and nucleus, respectively. **h** Competing endogenous RNA network of circGLS2. The edges were color-coded and proportional to the number of experimental data listed in the miRTarBase database. **i** Western blot assay of PTEN and AKT. **j** The IC_50_ assay for sorafenib upon circGLS2 knockdown or overexpression in HepG2 cells. **k** A proposed model for circGLS2’s regulatory mechanism. The dotted arrow represents a possible and/or indirect regulation relationship. AT adjacent tissues; CI confidence interval; FC fold change; HCC hepatocellular carcinoma; HR hazard ratio; IC_50_, half-maximal inhibitory concentration; T tumors; The “si-circGLS2” was the mixture of siRNAs; *P value < 0.05; **P value < 0.01; ***P value < 1 × 10^−3^; ****P value < 1 × 10^−4^

Next, we compared the expression levels of circRNAs between tumors and adjacent tissues and between PT and NRT. Overall, 53 of 2204 differentially expressed circRNAs overlapping between the two sets were considered as circRNA candidates closely associated with HCC recurrence (Supplementary Fig. S2a). A new circRNA circGLS2 was selected due to the higher changes in the both sets (Supplementary Fig. S2a, b). The sequencing data and experiments confirmed that circGLS2 is a *de novo* genuine circRNA transcribed from three exons of glutaminase 2 (GLS2, the human liver-type glutaminase) and then back-spliced into a circular structure (Fig. 1b). GSEA suggested that circGLS2 was highly associated with HCC (Supplementary Table S4). The Cox regression analyses in our 110-patient cohort indicated that circGLS2 expression in tumors was independently associated with a favorable RFS in HCC (Fig. 1c, Supplementary Table S5). Kaplan–Meier analysis confirmed these findings (Supplementary Fig. S2c, d). Thus circGLS2 functions as a strong negative regulator of HCC recurrence.

The expression of linear GLS2 was strongly correlated with circGLS2 and significantly decreased in tumors (Supplementary Fig. S3a, b). To elucidate the transcriptional regulation of GLS2, we combined the evidences of binding probability, proximity to transcription start site, occurrence frequency, and expression changes and discovered that KLF4 possibly regulated circGLS2 expression (Supplementary Fig. S3c, d). Luciferase reporter assay and ChIP-qPCR demonstrated that KLF4 directly bound to the GLS2 promoter (Fig. 1d). circGLS2 level was elevated upon ectopic expression of KLF4 in HepG2 cells (Fig. 1e). KLF4 was significantly downregulated in HCCs compared to the adjacent tissues both in our data and in the TCGA database (Supplementary Fig. S3e). Survival analysis suggested that KLF4 was associated with favorable RFS (Supplementary Fig. S3f). Thus, KLF4 is a key *trans*-activator of human GLS2 and circGLS2.

Using expression profiles of 371 tumors in the TCGA database, we built immunograms to visualize their tumor microenvironment (Supplementary Fig. S4a). Through unsupervised clustering, 86 immunologically cold tumors, characterized by the lack of T cell infiltration, and 62 hot ones rich in T cell infiltration were identified. Surprisingly, KLF4 was significantly upregulated in the hot tumors and the groups with higher immunogram scores (Supplementary Fig. S4b). Similar results were obtained in an independent dataset of 117 tumors from the GEO database (Supplementary Fig. S4c, d), suggesting that our conclusion is solid.

The top-ranked gene sets suggested that circGLS2 probably affected the pathways of cell proliferation, cell cycle, apoptosis, cell migration, and EMT (Supplementary Table S4). The transfections with circGLS2 siRNAs or circGLS2-overexpressing plasmids, as validated by qRT-PCR (Supplementary Fig. S5), indicated that circGLS2 inhibited the proliferation, colony formation, migration and promoted apoptosis of HCC cells (Supplementary Fig. S6). Western blot showed that fibronectin was greatly downregulated by overexpressing circGLS2 and upregulated in the cells with circGLS2 knockdown after its overexpression. Vimentin and β-catenin were upregulated by knocking down circGLS2 and downregulated by transfecting circGLS2, especially in Huh7 cells (Supplementary Fig. S7, S8a-c).

We took two steps to evaluate the therapeutic effects of circGLS2 on HCC xenograft in Nod-SCID mice: (i) Huh7 cells overexpressing circGLS2 in lentivirus were subcutaneously transplanted into Nod-SCID mice; (ii) circGLS2 siRNAs were intratumorally injected to treat HCC tumors subcutaneously transplanted with Huh7 cells. The tumors overexpressing circGLS2 were significantly smaller than those with empty lentivirus. The tumors injected with circGLS2 siRNAs were significantly bigger than the controls (Fig. 1f and Supplementary Fig. S8d). Immunochemistry indicated that fibronectin, vimentin, and β-catenin were upregulated in the tumors of Huh7 cells with circGLS2 knockdown (Supplementary Fig. S8e, f). Thus, circGLS2 suppressed tumor progression at least partially via inhibiting EMT.

Subcellular localization assay indicated that circGLS2 concentrated in the cytoplasm and nucleus of the parental and circGLS2-overexpressing Huh7 cells (Fig. 1g). Our RNA pulldown assay found six differentially expressed miRNAs bound to circGLS2 (Supplementary Fig. S9a and Table S6). After filtering the miRNA–target axes with weak experimental evidence and excluding hsa-miR-7706 sharing no nodes with other miRNAs, a competing endogenous RNA network was constructed (Fig. 1h). GSEA revealed that the five miRNAs overlapped 33 gene sets with circGLS2, indicating a strong functional connection (Supplementary Table S7).

Among the five miRNAs, hsa-miR-222-3p had the most intensive binding signal on the circGLS2-specific probe (Supplementary Fig. S9a). Hsa-miR-222-3p was upregulated in the tumors from the TCGA database (Supplementary Fig. S9b), which is consistent with our data (FC = 2.12, FDR = 0.016). Both GSEA and Kaplan–Meier analyses suggested that hsa-miR-222-3p was associated with unfavorable prognosis (Supplementary Fig. S9c and Table S7). The reporter assay suggested that circGLS2 served as a sponge for hsa-miR-222-3p (Supplementary Fig. S9d). Hsa-miR-222-3p increased the proliferation, migration, and colony formation of HepG2 cells (Supplementary Fig. S9e-g). The proliferation was attenuated by overexpressing circGLS2, which was partially restored by co-transfection of hsa-miR-222-3p (Supplementary Fig. S9e).

Our network indicated that PTEN was the most outstanding hub node linked to the miRNAs. The miR-222-3p–PTEN axis was strongly evident (Fig. 1h). Western blot assay indicated that PTEN was downregulated after knocking down circGLS2 while upregulated upon overexpression, quite in contrast to phosphorylated-AKT (p-AKT) (Ser473) (Fig. 1i). KLF4 and GLS2 were significantly downregulated, whereas fibronectin was upregulated in sorafenib-resistant Huh7 cells (Supplementary Fig. S10a). KLF4 was also downregulated in two of the sorafenib-resistant HCC organoids of GSE182593 (Supplementary Fig. S10b).^5^ Moreover, according to the BIOSTORM data, the transcription level of fibronectin was significantly downregulated in the sorafenib-treated HCC patients with better RFS (*i.e*., responder) (Supplementary Fig. S10c). IC_50_ values of sorafenib were significantly decreased upon circGLS2 overexpression and increased after circGLS2 knockdown in all three cell lines (Fig. 1j and Supplementary Fig. S10d). The expression of circGLS2 was significantly lower in the sorafenib-resistant HCC organoid than in the parental organoid (Supplementary Fig. S10e). The growths of the parental and sorafenib-resistant HCC organoids were significantly suppressed by ectopic expression of circGLS2 (Supplementary Fig. S10f).Thus, circGLS2 increases the sensitivity of HCC cells to sorafenib.

In conclusion, recurrent HCC enriches gene sets representing unfavorable prognosis, compared to non-recurrent HCCs. However, recurrent HCC is less malignant than their primary counterparts, possibly because of postoperative antiviral treatments. circGLS2, a new circRNA *trans*-activated by an anti-inflammatory and anti-EMT factor KLF4, is a key HCC recurrence suppressor. circGLS2 functions by attenuating the AKT activation via regulating hsa-miR-222-3p-PTEN signaling, thereby enhancing HCC’s sensitivity to sorafenib. circGLS2 should be a novel therapeutic agent for recurrent HCC (Fig. 1k).

## Supplementary information

Supplementary Materials

Supplementary Figures

Supplementary Table S3

Supplementary Table S4

## Data Availability

All data generated or analyzed during this study are included either in this article or in the supplementary information files. The data of circRNA-Seq, RNA-Seq, small RNA sequencing, and RNA pulldown were deposited in the Gene Expression Omnibus (GEO) database with the accession GSE169391. The RNA-Seq data of HCC organoids was deposited in the GEO database with the accession GSE182593.
