# Supplementary material for: circGLS2 inhibits hepatocellular carcinoma recurrence via regulating hsa-miR-222-3p–PTEN–AKT signaling
Source: Signal Transduct Target Ther. 2023 Feb 17;8:67. doi: 10.1038/s41392-022-01275-6 (PMC9935627; doi:10.1038/s41392-022-01275-6)
Supplement: Supplementary file 1 — Supplementary Materials [file 41392_2022_1275_MOESM1_ESM.docx]

Supplementary Materials for

**circGLS2 inhibits hepatocellular carcinoma recurrence by attenuating AKT/β-catenin signaling via regulating hsa-miR-222-3p-PTEN axis**

Xi Chen^1,3^, Ting Wu^1,3^, Linfeng Xian^1,3^, Longteng Ma^1,3^, Nan Li^2^, Wenbin Liu^1^, Peng Cai^1^, Xiaojie Tan^1^, Jianhua Yin^1,^ ✉, Guangwen Cao^1,^ ✉

^1^ Department of Epidemiology, Second Military Medical University, Shanghai, P R China; ^2^Department of Surgery, Eastern Hepatobiliary Surgery Hospital, Second Military Medical University, Shanghai, P R China; ^3^These authors contributed equally.

**Correspondence to:** Jianhua Yin, [hawkyjh163@163.com](mailto:hawkyjh163@163.com); Guangwen Cao, [gcao@smmu.edu.cn](mailto:gcao@smmu.edu.cn).

**This PDF file includes:**

Materials and Methods

Supplementary Text

Figures S1 to S10

Tables S1, S2, S5, S6, and S7

**Materials and Methods**

HCC patients and clinical samples

Thirteen pathologically diagnosed HCC patients (four recurred within two years after curative resection and received a second radical resection; nine did not recur five years after the resection) were enrolled into the finding cohort. Another 110 pathologically confirmed HCC patients were enrolled into a validation cohort. These patients received radical resection at the Eastern Hepatobiliary Surgery Hospital (Shanghai, China) during February 2011 and September 2012. All enrolled patients were seropositive for hepatitis B surface antigen and HBV DNA, had not received any previous medical treatments, had a 2-cm surgical margin without intrahepatic and distant metastasis, and received lifetime antiviral treatment with nucleotide/nucleoside analogs after curative surgery. Tumor tissues, paired recurrent tumor tissues, and paired adjacent tissues were removed and stored at −80°C immediately after surgery. The study protocol conformed to the 1975 Declaration of Helsinki and was approved by the ethics committee of the Second Military Medical University and the ethics committee of Eastern Hepatobiliary Surgery Hospital (No.2019OE-023). A signed informed consent was obtained from each participant.

Preparation of tissue RNA

Total RNA was extracted from tumor tissues and the corresponding adjacent liver tissues using mirVana miRNA isolation Kit (Ambion, Austin, TX) and quantitated using the Nanodrop 2000 (Thermo Fisher, Waltham, MA). RNA integrity was assessed by Agilent 2100 Bioanalyzer (Agilent Technology, Santa Clara, CA). Aliquots of each qualified total RNA sample were applied to construct three sequencing libraries.

circRNA sequencing and analyses

circRNA sequencing libraries were constructed using TruSeq Stranded Total RNA with Ribo-Zero Gold (Illumina, San Diego, CA) and Ribonuclease R (Lucigen, Middleton, WI). Then, the libraries were sequenced on the HiSeqTM 2500 sequencing platform (Illumina, San Diego, CA) and 150 bp paired-end reads were generated. CIRI software was applied to identify the circRNAs^1^. Reads spanning over back-splicing junction sites were collected to quantify all the circRNAs. As a measure of quality control, circRNAs with CPM > 1 occurring in more than 10 samples were kept for further analyses. Differentially expressed circRNAs were obtained using the edgeR package^2^. circRNAs with fold change (FC) ≥ 2 and false discovery rate (FDR) < 0.05 were considered as differentially expressed candidates.

Small RNA sequencing and analyses

Small RNA libraries were constructed using TruSeq Small RNA Sample Prep Kits (Illumina, San Diego, CA). The libraries were sequenced using the Illumina HiSeq X Ten platform and 150-bp paired-end reads were generated. The reads shorter than 15 nt or longer than 41 nt were also filtered. Adapters of reads were removed by cutadapt package^3^. Read pairs were mapped by Burrows–Wheeler Alignment tool (BWA) with parameters “-n 1 -o 0 -e 0 -l 8 -k 0”^4^. If two ends of a read pair were both mapped and their edit distance was less than 5 nt, then they were merged into one fragment. The overlap bases were determined by maximal Phred scores. These fragments were filtered according to their lengths by the same standard as mentioned above. The merged pair-end reads were mapped by BWA with the same parameter again. HTSeq was applied to count the read numbers of mature miRNAs in miRBase v22 (<http://www.mirbase.org/>)^5^. The differential miRNAs were determined by edgeR as aforementioned.

Transcriptome sequencing and analyses

Transcriptome sequencing libraries were constructed using TruSeq Stranded Total RNA with Ribo-Zero Gold and then sequenced on the Illumina HiSeq 2500. Reads with 150bp paired-ends were generated. The abundances of transcripts were estimated via performing eXpress on the reference transcripts of NCBI (GRCh38)^6^.

Function prediction for circGLS2/miRNAs and mining gene signatures enriched between different tumor types

GSEA was applied to determine the gene signatures enriched between different types of tumors and predicted the biological functions of circGLS2 and miRNAs^7^. For comparison between tumor types (e.g. PT *vs.* NRT), logarithmic fold changes were used as the metric to rank all genes. The “Prerank” method in the GSEA software was applied. Gene sets of chemical and genetic perturbations in MsigDB (http://software.broadinstitute.org/gsea/index.jsp) were randomized for 1000 times. For biological function prediction, circGLS2 (or miRNA) was used as a profile and pairwise Pearson correlation for each protein-coding gene was calculated. All these Pearson correlation coefficients were used for gene ranking. The rest steps were identical to those aforementioned. Gene sets with familywise error (FWER) < 0.05 were considered significantly enriched unless explicitly stated otherwise.

Transcription factor binding sites (TFBSs) analysis

The MotEvo-predicted TFBSs within the GLS2’s promoter were downloaded from SwissRegulon website (https://swissregulon.unibas.ch/sr/)^8, 9^. The hg19 genomic coordinates were converted to the hg38 ones by the liftover tool (https://genome.ucsc.edu/cgi-bin/hgLiftOver). TFBSs with posterior probabilities > 0.8 were kept for further analysis. The expression profiles of these transcription factors were retrieved from the TCGA database (https://xenabrowser.net/) to validate the expression changes in our data.

RNA pulldown assay and competing endogenous RNA (ceRNA) network construction

In total, 1 × 10^8^ HepG2 cells were collected, washed twice with RNA-free PBS, lysed, and centrifuged at 14,000 rpm for 15 min to collect the supernatant. circGLS2 or antisense RNA (control probe reverse-complementary to circGLS2) was labeled with biotin and incubated with Pierce Streptavidin Magnetic Beads (Thermo Fisher, Newton, MA) at 25 °C for 1 h. The probes are: circGLS2-specific probe, 5'-Biotin-CCAGCTGAGGGATGTAGGCTGCCACCTTGATCA GGGAGCTGACAACAA-3'; control probe, 5'-Biotin-TTGTTGTCAGCTCCCTGATCAAGGT GGCAGCCTACATCCCTCAGCTGG-3'. Cell lysate with circGLS2 probe-bound beads were incubated overnight at 4 °C. After washing with wash buffer, the RNA mix bound to the beads was eluted and extracted with RNeasy Mini Kit (Qiagen, Hilden, Germany) for the miRNA sequencing analysis. The miRNA sequencing library was prepared using NEBNext Multiplex Small RNA Library Prep Set for Illumina (New England Biolabs, Ipswich, MA). The libraries were denatured as single-stranded DNA molecules, captured on Illumina flow cells, amplified *in situ* as clusters, and finally sequenced for 50 cycles on Illumina HiSeq4000 sequencer.

The quality control procedure for the sequencing data obtained from RNA pulldown assay was identical to that of small RNA sequencing. BWA and HTSeq were utilized for read mapping and counting as aforementioned. CPMs were calculated to estimate miRNA levels. The ratios between the circGLS2-specific CPMs (adding 1 to take into account small values) and those of the control probe were calculated to represent miRNA enrichment levels. miRNAs with enrichment levels ≥ 3.5 and significantly upregulated in the tumors were collected.

The axes recorded in the miRTarBase 8.0 were queried and the records marked with weak experimental evidence were filtered^10^. The target genes remained along with circGLS2 and five miRNAs were selected to construct a ceRNA network. The number of experimental evidence for each axis was summarized and utilized to plot the edges in the ceRNA network.

Immunogram analyses

Eight-axis immunogram was built using data of 371 tumors in TCGA as previously reported^11^. Gene set variation analysis (GSVA) was performed to estimate the immunogram scores for each axis^12^. All tumors were equally classified into two groups according to the median value of each axis. By hierarchical clustering (Euclidean distance, Ward’s method), immunologically cold and hot tumors were visually determined. The expression changes of KLF4 were estimated in the groups of axes and between cold and hot tumors. To validate the KLF4’s expression changes, extra five transcriptome data sets were retrieved from Sequence Read Archive (SRA) database (listed below). Salmon^13^ was performed to obtain read count matrix. The potential batch effect among different studies was adjusted by the Combat-Seq method^14^.

**Five data sets collected to build the extra immunogram**

| **SRA accession** | **GEO accession** | **#Tumor** | **PMID** |
| --- | --- | --- | --- |
| SRP062885 | NA | 16 | 26867494 |
| SRP069212 | GSE77509 | 20 | 28194035 |
| SRP099053 | GSE94660 | 21 | 29212479 |
| SRP120360 | GSE105130 | 25 | 31429776 |
| SRP174991 | GSE124535 | 35 | 30814741 |

Abbreviation: GEO, gene expression omnibus; NA, not available; SRA, sequence read archive.

Validation of the circGLS2’s structure

Total RNA was extracted from tumor and paired adjacent tissues and treated with RNase R. Real-time quantification PCR was performed to quantify the abundance levels of circGLS2 and its linear products (NM_001280796). The primers are: circGLS2 forward, 5’-TTGTGGGCAAAGAGCCAAGT-3’, circGLS2 reverse, 5’-GATGTAGGCTGCCACCTTGA-3’; GLS2 forward, 5’-CTGCACTAAAGGCCACTGGA-3’, GLS2 reverse, 5’-TTGCTGCTCACAC ACTTTCG-3’.

Cell culture and transfection

Human HCC cell lines (HepG2, Huh7, and SK-Hep-1) and 293T cell line were purchased from the Cell Center of Shanghai Institute of Biological Sciences (Shanghai, China). Before the experiments, all cell lines were authenticated via genotyping short tandem repeat (STR) (Biowing, Shanghai, China). All cell cultures were tested for mycoplasma contamination every three months. All cell lines were cultured in DMEM/high glucose medium (HyClone, Logan, UT), supplemented with 10% fetal bovine serum (HyClone). circGLS2 sequence was routinely inserted into the PLCDH-ciR vector via EcoRI and BamHI restriction endonuclease sites. pLP1, pLP2, pLP-VSV-G, and PLCDH-ciR-circGLS2 were transfected into 293T cell line with Lipofectamine 3000 (Thermo Fisher). The lentivirus was concentrated by Lenti-X Concentrator (TaKaRa, Shiga, Japan) and then used to infect all HCC cell lines. The stable strains were screened by 2 µg/mL puromycin dihydrochloride (Thermo Fisher). The sequences of siRNAs for knocking down circGLS2 are: siRNA-1-sense, 5’-CUCCCUGAUCAAGGUGGCATT-3’, siRNA-1-antisense, 5’-UGCCACCU UGAUCAGGGAGTT-3’; siRNA-2-sense, 5’-GAUCAAGGUGGCAGCCUACTT-3’, siRNA-2-antisense, 5’-GUAGGCUGCCACCUUGAUCTT-3’.

qRT-PCR

Total cellular RNA was extracted by Trizol reagent (Invitrogen, Carlsbad, CA). The abundance of circGLS2 or NM_001280796 was quantified using PrimeScript RT Master Mix (TaKaRa). Hsa-miR-222-3p was quantified using Mir-X miRNA First-Strand Synthesis Kit (TaKaRa).

ChIP assay

ChIP was conducted using ChIP-IT Express Enzymatic Kit (Active Motif, Carlsbad, CA). HepG2 cells were transfected with pSLenti-EF1-mCherry-KLF4 (Obio, Shanghai, China) or the vector plasmid. Parental and KLF4-overexpressing HepG2 cells were subjected to the ChIP assay. Chromatin fragments were immunoprecipitated with antibody against KLF4 (ab10669, Abcam, Cambridge, MA). The rabbit IgG (#2729, Cell Signaling Technology (CST), Danvers, MA) served as the non-specific antibody control. The total (input) and ChIP-enriched DNA were subjected to quantitative PCR (qPCR). The enrichment value was calculated as a percentage of input. The primers are: forward, 5’-CCGTCTTGCTCTCATTGG-3’; reverse, 5’-GGCTTTCTAGTGGGTG GA-3’.

Fluorescent In Situ Hybridization (FISH)

For RNA FISH, the Ribo^TM^ FISH kit (RiboBio, Guangzhou, China) was applied to identify the cellular location of circGLS2 in Huh7 cells. Cells were fixed in 0.5% Triton X-100 (Beyotime, Shanghai, China) in paraformaldehyde for 30 min at 25 °C. Cy3-labeled GLS2, 18S-RNA, and U6-RNA were incubated overnight at 37 °C in the dark and washed in SSC (Beyotime) three times at 42 °C. DNA stained with DAPI for 10 min at 25 °C was washed in PBS. Dish (35 mm, Ibidi, Martinsried, Germany) was examined by confocal fluorescence microscopy (Leica).

Cell proliferation assay

Cell proliferation assay was performed using CCK-8 Kit (DoJinDo, Osaka, Japan). First, 1000–1500 cells were seeded into each well of 96-well plates. Then, 100 µL of DMEM (10% CCK-8) was added, and the cells were incubated for 2 h. The number of viable cells was estimated by optical density (OD_450_) measurements every 24 h.

Migration assay

Cell migration ability was measured by using a 6.5 mm Transwell with 8.0 µm Pore Polycarbonate Membrane insert (Corning, New York, NY). The bottom chamber was filled with DMEM (10% FBS). In total, 10^4^ cells were placed in upper wells with 400 µL of serum-free DMEM (2% BSA). The cells were incubated for 16 h at 37 °C in 5% CO_2_, followed by crystal violet dyeing for 15 min. Then, the images were taken.

Apoptosis and cell cycle assay

Flow cytometry (BECKMAN, Pasadena, CA) was applied for apoptosis and cell cycle analysis. Cells were rinsed with PBS. Then, 10^6^ cells were suspended in 500 µL PBS with 50 µL 10× Binding Buffer, 5 µL APC AnnexinV (BD, New York, NY), and 5 µL 7-AAD (BD). For cell cycle analysis, rinsed cells were suspended in 500 µL precooled 70% ethanol and fixed in −20 °C overnight. The fixed cells were collected, and 200 µL of PI/DNase Staining buffer (BD) was added. Apoptosis and cell cycle were analyzed after incubation at 25 °C for 15 min.

Cell colony formation assay

The transfected cells were seeded into 6-well plates at a density of 2,000 cells per well. After being cultured at 37 °C in a 5% CO_2_ incubator for two weeks, the cells were fixed with methanol and stained with 0.1% crystal violet. Finally, the colonies were imaged and counted.

Animal research

All animal care and experimental procedures were conducted according to the guidelines of the National Institutes of Health and were approved by the Institutional Animal Care and Use Committee of Second Military Medical University. Male, four-week-old Nod-SCID mice purchased from Jihui laboratory animal care cooperation (Shanghai, China) were randomly divided into four groups (8 mice per group, 2 standby mice were also added in a group). Then, 1.5 × 10^6^ Huh7 cells infected with recombinant lentivirus overexpressing circGLS2 or the same amount of parental cells infected with lentivirus negative control were mixed with Matrigel and *s.c*. injected into Nod-SCID mice, respectively. In another two groups, 1.5 × 10^6^ Huh7 cells were *s.c*. transplanted into Nod-SCID mice. On the 10^th^, 13^th^, 16^th^, and 19^th^ day after the implantation, siRNAs against circGLS2 or the siRNA with scramble sequence were intratumorally injected. Tumor growth was measured every other day. Tumor volume was calculated with the formula: V = length × width^2^/2. Mice were sacrificed after three weeks, and the tumors were dissected for weight and size measurements and immunohistochemistry.

Western blot and immunohistochemistry

Cells were lysed on ice in Pierce RIPA Buffer containing Halt Protease Inhibitor Cocktail (Thermo Fisher). Protein concentration was detected with Quick Start Bradford 1× Dye Reagent (Bio-Rad, Hercules, CA). The blocked protein was incubated with the primary antibodies of E-cadherin (#3195S, CST), N-cadherin (#13116S, CST), vimentin (#5741T, CST), β-catenin (#8480S, CST), fibronectin (#26836S, CST) (Epithelial–Mesenchymal Transition Antibody Sampler Kit, 1:1000), snail (#3879S, CST), and GAPDH antibody (#3683S, CST) at 4 °C overnight. The blocked protein was washed with TBST three times and incubated with the HRP-linked antibody (anti-rabbit IgG, 1:2000, #7074S, CST) for 1 h. The blots were visualized by Immobilon Western Chemiluminescent HRP Substrate (Merck millipore, Darmstadt, Hessen, Germany).

For immunohistochemistry (IHC), tissue samples were fixed in 4% paraformaldehyde and embedded in paraffin. The tissues were sectioned and incubated with the primary antibodies of E-cadherin, N-cadherin, vimentin, β-catenin, fibronectin, and GAPDH antibody at 4 °C overnight. The HRP-linked antibody was incubated at 37 °C for 1 h.

Luciferase reporter assay

To determine the regulation of GLS2 expression by KLF4, the promoter region (1kbp upstream) of GLS2 was synthesized and cloned into pGL4.10 plasmid (Obio Technology). This plasmid was then transfected into 293T cells for luciferase reporter assay. To validate the binding between circGLS2 and hsa-miR-222-3p, the sequence of circGLS2 was cloned into the H306 pMIR-REPORT luciferase vector. The cells were co-transfected with the reporter plasmid and hsa-miR-222-3p or mimics using the Lipofectamine 3000 transfection reagent. The luciferase activity was quantified at 48 h with a dual-luciferase reporter assay (Promega, Madison, WI).

Sorafenib resistance analyses

The original data of GSE140202 consisting of sorafenib-sensitive and -resistant Huh7 cell lines was retrieved from SRA database. Salmon was performed to obtain gene expression data. The BIOSTORM microarray data (GSE109211) was retrieved from Gene Expression Omnibus (GEO) database. The data consisted of 73 HCC patients treated with placebo and 67 treated with sorafenib. Among these 67 patients, 21 had better recurrence-free survivals (RFSs) (responders), while the remaining 46 patients had unfavorable outcomes (non-responders).

The HCC organoids were constructed as previously reported^15^. Total RNA was extracted using TRIzol (Invitrogen). The libraries were constructed using TruSeq Stranded Total RNA with Ribo-Zero Gold (Illumina, San Diego, CA) and then sequenced on the Illumina NovaSeq 6000 sequencing platform (Illumina, San Diego, CA). Reads with 150 bp paired-ends were generated. The abundances of transcripts were estimated via performing eXpress on the reference transcripts of NCBI (GRCh38)^16^.

The half-maximal inhibitory concentrations (IC50) values were determined in three cell lines upon knockdown and over-expression of circGLS2. Cell medium containing 10^6^ cells/mL was dripped into a 96-well plate (100 μL medium / well). The plate was incubated for 16 h for cell adhesion. Cells were cultured in the medium containing different concentrations of sorafenib for 24 h. Then 100μL DMEM containing 10% CCK8 was added into each well and incubated for 2 h at 37 °C. The number of viable cells was measured using OD_450_. The cell index at each concentration was estimated as previously described^17^. The inhibition rate was calculated as 1 - cell index. The “drc” package^18^ was used to perform the IC50 curves fitting and differential tests.

Statistical analysis

The differences between two or multiple groups were determined using the t-test or ANOVA. A Cox proportional hazard model was conducted to calculate hazard ratio (HR) and 95% confidence interval (CI) for each variable. Significant variables in the univariate Cox analysis were introduced into the multivariate Cox model, and backward stepwise Wald method was applied to determine the factors that independently contributed to postoperative prognosis. Kaplan–Meier analysis was conducted to estimate OS and DFS, and log-rank test was applied to compare the difference between the two groups. During the process, a threshold was chosen from 1,000 possible thresholds from the minimal expression level to the maximal. Every *p* value of the log-rank test was calculated. The test providing the smallest *p* value was selected and the corresponding threshold was considered optimal. False discovery rate was calculated using the Benjamini–Hochberg method. All statistical tests were two-sided and performed using R platform (v4.0.2) and GraphPad Prism v8.4.0 (GraphPad Software, San Diego, CA). The threshold of α was 0.05 except when explicitly stated.

**Supplementary Text**

*Comparison among three types of tumors by transcriptome, circRNA, and miRNA data*

There were three types of tumors in our data: non-recurrent tumors (NRT), primary tumors (PT), and recurrent tumors (RT) as illustrated in Fig. 1a. Their paired adjacent tissues were also collected as controls. In Gene set enrichment analysis (GSEA), few gene sets were enriched among the three types of adjacent liver tissues, indicating that the expression patterns in adjacent liver tissues were concordant with each other (Supplementary Fig. S1b, top three panels). By comparison, GSEA among the three tumor types obtained evidently more enriched gene sets (97 between PT and NRT; 88 between RT and NRT; and 49 between RT and PT) (Supplementary Fig. S1b, bottom three panels and Supplementary Table S3). In the top enriched gene sets between PT and NRT, HCC recurrence- and tumorigenesis-related gene sets were discovered (Supplementary Fig. S1c, left). Similar gene sets were also identified between RT and NRT (Supplementary Fig. S1c, middle). Thus, PT and RT were more malignant than NRT. Interestingly, gene sets enriched between RT and PT suggested that recurrent HCCs were more benign than primary HCCs (Supplementary Fig. S1c, right). (Note the change of gene set suffixes.)

In total, 59,711 circRNAs were identified and 55,752 (93.4%) of them were annotated by the circAtlas database (<http://circatlas.biols.ac.cn/>). circRNAs with low expression levels were filtered, leaving 11,432 circRNAs for further analyses. Of 2,434 circRNAs with fold changes (FC) ≥ 2 both between all tumors and adjacent tissues and between RTs and PTs, 1,645 (67.6%) were up- or down-regulated in the RTs compared to PTs while reversely regulated in the tumors compared to adjacent tissues (Supplementary Fig. S1d, top). Since the tumors were more malignant than the paired adjacent tissues, it could be deduced that RTs were more benign than PTs, concordant with the results of GSEA. Similar phenomenon was also observed in the miRNA data (Supplementary Fig. S1d, bottom). Besides, the results in the both circRNA and miRNA data also suggested that PTs were more malignant than NRTs (Supplementary Fig. S1e).

*Identification of circGLS2, an HCC recurrence-related circRNA*

We compared the expression levels of circRNAs between tumors and adjacent tissues, which obtained 2,204 differentially expressed circRNAs. Then, 66 differentially expressed circRNAs between PT and NRT were also obtained by the same procedure. In total, 53 circRNAs overlapping between the two sets were considered as circRNA candidates closely associated with HCC recurrence (Supplementary Fig. S2a, left). Of those, a new circRNA named circGLS2 was selected due to the higher changes between tumors and adjacent tissues (fold change [FC] = −51.13, false discovery rate [FDR] = 3.21 × 10^-13^) and between PT and NRT (FC = −388.35, FDR=4.16 × 10^-5^) (Supplementary Fig. S2a, right and S2b, left). Our data show that circGLS2 has 116.79 back-splicing junction reads on average, which is a strong signal indicating that circGLS2 is a bona fide circRNA (Supplementary Fig. S2a, right). The sequencing data indicate that circGLS2 might be transcribed from three exons of glutaminase 2 (GLS2, the human liver-type glutaminase) and then back-spliced into a circular structure. We then designed circGLS2-specific divergent primers and amplified and sequenced circGLS2 in the tumors and adjacent liver tissues. The back-splicing junction site was clearly observed (Supplementary Fig. 1b, left). After digested with RNase R, circGLS2 showed little change, while GLS2 decreased significantly in the HCC tissues (Supplementary Fig. 1b, right). Thus, circGLS2 is a *de novo* genuine circRNA. Next, we quantified circGLS2 in 110 pairs of tumors and adjacent tissues from an independent cohort of our HCC patients. The expression of circGLS2 was significantly lower in tumors than in adjacent liver tissues (*p* = 2.95 × 10^-9^) and in tumors from recurrent HCC patients than in tumors from HCC patients without recurrence (*p* = 6.83 × 10^-3^) (Supplementary Fig. S2b, middle and right). GSEA was conducted to predict the biological functions of circGLS2, and 261 gene sets were significantly enriched (Supplementary Table S4). Surprisingly, of the top 20 enriched gene sets ranked by normalized enrichment scores (NES), 17 (85%) were literally HCC or liver gene signatures. Especially, two gene sets suggested that circGLS2 predicted favorable recurrence-free survival (RFS) and overall survival (OS) (WOO_LIVER_CANCER_RECURRENCE_DN, NES = 3.13, familywise error rate (FWER) < 1e-3; LEE_LIVER_CANCER_SURVIVAL_UP, NES = 3.10, FWER < 1e-3) (Supplementary Table S4). Kaplan–Meier survival analysis in our 110-patient cohort indicated that low circGLS2 expression in the tumors was significantly associated with unfavorable prognosis (*p* = 9.46 × 10 ^-3^ for OS and *p* = 2.64 × 10 ^-5^ for RFS) (Supplementary Fig. S2c). We also performed survival analysis using circGLS2 expression in the tumors adjusted for the expression in the paired adjacent tissues. The result was consistent with the above observations (Supplementary Fig. S2d). Thirteen variables listed in the Table S2 were included in the univariate Cox regression analyses. The ascites, portal vein tumor thrombi, and tumor size were excluded because there were not sufficient patients in the negative (or positive) group (Supplementary Table S2). The result suggested that multiple tumors, microscopic vascular invasion, and microsatellite increased the risk of postoperative recurrence, whereas circGLS2 decreased the risk (Supplementary Table S5). The multivariate Cox regression analyses indicated that circGLS2 was independently associated with a favorable RFS in HCC (Fig. 1c). This line of evidence indicates that circGLS2 functions as a strong negative regulator of HCC recurrence and predicts a favorable prognosis.

*Therapeutic potential of circGLS2 on HCC cell proliferation and HCC xenograft*

The top ranked gene sets enriched by circGLS2 suggested that it probably affected the pathways of cell proliferation, cell cycle, and apoptosis (Supplementary Table S4). As the gene sets of metastasis and EMT were enriched, circGLS2 might also regulate cell migration and EMT. The effects of transfections with siRNAs against circGLS2 or circGLS2-overexpressing plasmids on the expression of circGLS2 in HepG2, Huh7, and SK-Hep-1 cells were validated by real-time quantitative reverse transcription PCR (qRT-PCR) (Supplementary Fig. S5a and S5b). We quantified circGLS2 in four HCC cell lines and selected 3 (HepG2, Huh7 and SK-Hep-1) of them for further analyses. As circGLS2 was of tumor suppressive, the levels of circGLS2 expression were relatively lower in these cell lines (Supplementary Fig. S5c). Cell Counting Kit-8 (CCK-8) assay showed that the proliferations of HepG2 and SK-Hep-1 cells were significantly inhibited by overexpressing circGLS2 and significantly enhanced by knocking down circGLS2 in HepG2 cells (Supplementary Fig. S6a). The colony formation ability of SK-Hep-1 cells was significantly inhibited by overexpressing circGLS2 and significantly enhanced by knocking down circGLS2 (Supplementary Fig. S6b). The migration ability of SK-Hep-1 and Huh7 cells was evidently attenuated by overexpressing circGLS2 and elevated by knocking down circGLS2 (Supplementary Fig. S6c, S6d). We also found that apoptotic rates of HepG2 and Huh7 cells were significantly increased by overexpressing circGLS2 and decreased by knocking down circGLS2 (Supplementary Fig. S6e, S6f). The proportion of cells at the S phase was significantly decreased by overexpressing circGLS2 and increased by knocking down circGLS2 in Huh7 cells (Supplementary Fig. S6g). The endogenous mRNA levels of six EMT markers were measured by qRT-PCR (Supplementary Fig. S7). Then the abundance of six EMT markers in HepG2 and Huh7 cells was measured by Western blot. The level of fibronectin was greatly down-regulated by over-expressing circGLS2 and up-regulated in the cells with circGLS2 knockdown after its overexpression. The levels of vimentin and β-catenin were up-regulated by knocking down circGLS2 and down-regulated following the transfection of circGLS2, especially in Huh7 cells (Supplementary Fig. S8a, S8b, and S8c).

We took two steps to evaluate the therapeutic effects of circGLS2 on the growth of Huh7 xenograft in non-obese diabetic severe combined immunodeficiency (Nod-SCID) mice: (i) Huh7 cells overexpressing circGLS2 in recombinant lentivirus were subcutaneously (*s.c.*) transplanted into Nod-SCID mice; (ii) siRNAs against circGLS2 was intratumorally injected to treat HCC tumors *s.c.* transplanted with Huh7 cells. It was found that the tumors overexpressing circGLS2 were significantly smaller than those with empty lentivirus. Interestingly, the tumors injected with circGLS2 siRNAs were significantly bigger than the controls (Fig. 1f and Supplementary Fig. S8d). Immunochemistry indicated that the expression levels of fibronectin, vimentin, and β-catenin increased in the tumors derived from Huh7 cells with circGLS2 knockdown (Supplementary Fig. S8e, S8f). Thus, circGLS2 suppressed tumor progression, via partially inhibiting EMT.

*Discussion*

In this study, HCCs and adjacent liver tissues of non-recurrent HCC patients and recurrent HCC patients were applied for deep sequencing analyses of circRNA, miRNA, and mRNA expression profiles. All the high-throughput omics data were generated by state-of-the-art technology, and their library sizes met current standards. About 60k circRNAs were identified in our circRNA-seq data, and 93.4% of them were annotated by the circAtlas database, suggesting that our data are of high quality. We found that the significant differentially expressed circRNAs were more downregulated. The expression levels of these circRNAs were inversely associated with the occurrence (HCCs vs. adjacent liver tissues) and postoperative recurrence (recurrent HCC vs. non-recurrent HCCs). Formerly identified HCC suppressive circRNAs such as cSMARCA5 and circMTO1 by other teams were also included,^19, 20^ indicating our strategy works well. Of those differentially expressed circRNAs, circGLS2 was identified as a novel and the most potent tumor suppressor and recurrence inhibitor. Interestingly, some down-regulated circRNAs including cSMARCA5, circMTO1, circC3P1, circLARP4, and circADAMTS13 have been shown to suppress the growth and aggressiveness of HCC cells.^19, 20-23^ Thus, these downregulated circRNAs represent novel therapeutic candidates for the recurrence of HCC.

In this study, we demonstrated that both primary and recurrent tumors enriched the gene sets that indicate unfavorable prognosis in HCC. Moreover, the recurrent tumors showed more benign signature than did the primary tumors, while a homogeneous expression pattern was observed among those adjacent liver tissues. This result was also supported by the circRNA and miRNA data as well (Supplementary Fig. S1b-S1e). In this study, all involved patients were chronically infected with HBV and received lifetime antiviral treatments after curative surgery. Four patients with early recurrence were included. Early recurrence is mostly related to dissemination of primary HCC, rather than from *de novo* tumors arising from the “field effect” in diseased liver.^24^ In our previous study, antiviral treatment greatly improved the postoperative prognosis via decreasing hepatic inflammation.^25^ Decreased selection pressure in the tumor microenvironment with reduced inflammatory activity might decelerate HCC evolution after receiving postoperative antiviral treatment. Thus, the recurrent HCCs exhibit more benign signature than did the primary counterparts.

The mechanisms by which circGLS2 inhibits HCC progression were investigated in this study. The expression of linear GLS2 was strongly correlated with that of circGLS2. circGLS2 is transcribed from exons 5, 6, and 7 of GLS2 and then back-spliced into a circular structure. The transcription of circGLS2 and linear GLS2 should be under the transcriptional control of the same transcriptional regulatory sequences. GLS2 functions as an oncogene in luminal-subtype breast cancer,^26^ but it also inhibits migration and invasion of HCC cells by repressing EMT.^27^ Clearly, circGLS2 inherits the characteristics of its parental molecule as a tumor suppressor in HCC. Our luciferase reporter assay and ChIP-qPCR demonstrated that the expression of GLS2 was under the transcriptional regulation of KLF4, an evolutionarily conserved zinc finger-containing transcription factor. Interestingly, KLF4 was down-regulated in HCC and inversely associated with unfavorable RFS in HCC (Supplementary Fig. S3e and S3f), which is consistent with previous findings.^28, 29^ KLF4, one of four factors involved in the induction of pluripotent stem cells, functions as an anti-inflammatory factor and tumor suppressor.^30-32^ As an anti-EMT factor, KLF4 is regulated at both transcriptional and post-transcriptional levels.^33^ It has been proven that tumor necrosis factor receptor-associated factor 7, a component of the nuclear factor kappa B (NF-κB) pathway, degrades KLF4 protein through ubiquitin by interacting with its N-terminus, thus promoting HCC cell migration and invasion.^34^ NF-κB is one of the major inflammatory pathways driving the progression of HCC. Thus, HCC-promoting inflammatory factors might degrade KLF4 protein, resulting in decrease in the *trans*-activation of GLS2 gene in liver. The down-regulation of GLS2 transcription leads to reduced expression of circGLS2, thus facilitating the progression of HCC. Based on this line of evidence, we built the eight-axis immunograms from 371 tumors in the TCGA data and 117 tumors merged from the GEO data. The results both suggested that KLF4 was up-regulated in the HCC patients with increased immune cell infiltration (hot tumor) (Supplementary Fig. S4b, S4d).

We provided evidence from cell culture, animal experiment, and cohort study suggesting that circGLS2 functions as an HCC suppressor. circGLS2 inhibits the progression of HCC by inducing apoptosis and partially attenuating EMT. We also found that circGLS2 functioned via regulating a ceRNA network. circGLS2 regulated these target genes by sponging five miRNAs. The miRNAs are proven to promote HCC progression. miR-222-3p serves as a marker for a poor prognosis in HCC.^35^ miR-130b-3p contributes to HCC progression and angiogenesis.^36^ miR-18a-5p is overexpressed in HCC and associated with HCC prognosis.^37^ miR-10b-3p promotes HCC progression via targeting CMTM5.^38^ miR-106b-3p serves as a diagnostic biomarker of HCC.^39^ Our experiments confirmed the binding and interaction between circGLS2 and hsa-miR-222-3p. PTEN, an important tumor suppressor, was identified as a functional target of hsa-miR-222-3p. circGLS2 exerted its cancer suppressor function by hsa-miR-222-3p–PTEN axis (Fig. 1h). The phosphorylation of AKT (Ser473) and β-catenin are attenuated by this axis (Fig. 1i and Supplementary Fig. S8a-S8c). In our study, circGLS2 is proven to increase the sensitivity of the tumor cells to sorafenib (Fig. 1j and Supplementary Fig. S10d), which was predictable as AKT/β-catenin activation has been shown to increase the resistance of HCC cells to sorafenib.^40^ The expression of fibronectin was elevated upon circGLS2’s knockdown (Supplementary Fig. S8c), probably due to the activation of AKT. The activation of PI3K-AKT pathway mediates up-regulation of fibronectin, thus facilitating HCC metastasis.^41^ circGLS2’s knockdown plays a critical role in promoting HCC recurrence by inhibiting PTEN and activating AKT signaling, because the PTEN/AKT pathway is important in the progression of HCC.^42^ Fibronectin, transcriptionally down-regulated in the sorafenib responder of BIOSTROM clinical trial (Supplementary Fig. S10c), has been shown as a proteomic biomarker of invasive HCC. Taken together, circGLS2 has potential as a novel therapeutic agent to treat recurrent HCC.

Our animal study showed that the tumor of human HCC cells (Huh7) expressing circGLS2 grew much slower than the control while the injection of siRNA to circGLS2 greatly facilitated the growth of the HCC xenograft in Nod-SCID mice. These data indicate that circGLS2 might be an effective therapeutic agent for the treatment of HCC recurrence. However, circRNA gain-of-function cancer therapy via overexpressing natural or artificial cancer-suppressor circRNAs faces some technical challenges, such as difficulty in generating a large amount of circRNAs *in vitro*, unwanted immune system activation, poor efficiency of delivery of circRNA expression cassettes *in vivo*, and the methods in delivering circRNAs to cells.^43-45^ With rapid progresses in synthetic biology and advances in nanoparticle and exosome delivery systems, circRNA-based cancer therapy will become reality in near future.

To the best of our knowledge, this is the first study to identify HCC recurrence-related circRNAs among non-recurrent tumors, primary tumors, and paired recurrent tumors using multi-omics sequencing analyses of circRNome, miRNome, and transcriptome. The identified circRNAs are annotated and validated by state-of-the-art technology and traditional experiments at cell culture, animal model, and human population levels. However, our study has limitations. First, epigenetic silencing of KLF4 caused by inflammation was not investigated. Second, the mechanism by which circGLS2 was generated from its parent transcript remains to be identified. Third, the therapeutic effect of circGLS2 needs to be evaluated in different models.

In summary, we discovered that both primary and recurrent tumors showed more malignant gene expression pattern and enriched gene sets that indicate an unfavorable prognosis of HCC patients compared to non-recurrent tumors. Furthermore, the recurrent tumors showed more benign feature than did their primary tumors, possibly because of persistent postoperative antiviral treatments. We identified circGLS2, whose transcription was *trans*-activated by an anti-inflammatory and anti-EMT factor KLF4, as a key HCC recurrence suppressor. circGLS2 functions by attenuating the AKT/β-catenin activation via regulating hsa-miR-222-3p–PTEN signaling, thereby enhances to sensitivity of sorafenib. circGLS2 could be a biomarker to predict postoperative prognosis in HCC and functions as a novel therapeutic agent to treat recurrent HCC.

Reference

1. Gao, Y., Wang, J. & Zhao, F. CIRI: an efficient and unbiased algorithm for de novo circular RNA identification. *Genome Biol.* **16**, 4–19 (2015).
2. McCarthy, D. J., Chen, Y. & Smyth, G. K. Differential expression analysis of multifactor RNA-Seq experiments with respect to biological variation. *Nucleic Acids Res.* **40**, 4288–4297 (2012).
3. Martin, M. Cutadapt removes adapter sequences from high-throughput sequencing reads. *EMBnet j.* **17**, 10–12 (2011).
4. Li, H. & Durbin, R. Fast and accurate short read alignment with Burrows-Wheeler transform. *Bioinformatics* **25**, 1754–1760 (2009).
5. Anders, S., Pyl, P. T. & Huber, W. HTSeq--a Python framework to work with high-throughput sequencing data. *Bioinformatics* **31**, 166–169 (2015).
6. Roberts, A. & Pachter, L. Streaming fragment assignment for real-time analysis of sequencing experiments. *Nat. Methods* **10**, 71–73 (2013).
7. Subramanian, A. *et al.* Gene set enrichment analysis: a knowledge-based approach for interpreting genome-wide expression profiles. *Proc. Natl. Acad. Sci. U. S. A.* **102**, 15545–15550 (2005).
8. Arnold, P., Erb, I., Pachkov, M., Molina, N. & van Nimwegen, E. MotEvo: integrated Bayesian probabilistic methods for inferring regulatory sites and motifs on multiple alignments of DNA sequences. *Bioinformatics* **28**, 487–494 (2012).
9. Pachkov, M., Balwierz, P. J., Arnold, P., Ozonov, E. & van Nimwegen, E. SwissRegulon, a database of genome-wide annotations of regulatory sites: recent updates. *Nucleic Acids Res.* **41**, D214–D220 (2012).
10. Hsu, S.-D. *et al.* miRTarBase: a database curates experimentally validated microRNA–target interactions. *Nucleic Acids Res.* **39**, D163–D169 (2011).
11. Karasaki, T. *et al.* An Immunogram for the Cancer-Immunity Cycle: Towards Personalized Immunotherapy of Lung Cancer. *J. Thorac. Oncol.* **12**, 791–803 (2017).
12. Hänzelmann, S., Castelo, R. & Guinney, J. GSVA: gene set variation analysis for microarray and RNA-seq data. *BMC Bioinformatics* **14**, 7–21 (2013).
13. Patro, R., Duggal, G., Love, M. I., Irizarry, R. A. & Kingsford, C. Salmon provides fast and bias-aware quantification of transcript expression. *Nat. Methods* **14**, 417–419 (2017).
14. Zhang, Y., Parmigiani, G. & Johnson, W. E. ComBat-seq: batch effect adjustment for RNA-seq count data. *NAR Genom. Bioinform.* **2**, lqaa078 (2020).
15. Liu, D. *et al.* circKCNN2 suppresses the recurrence of hepatocellular carcinoma at least partially via regulating miR-520c-3p/methyl-DNA-binding domain protein 2 axis. *Clin. Transl. Med.* **12**, e662 (2022).
16. Trapnell, C. *et al.* Transcript assembly and quantification by RNA-Seq reveals unannotated transcripts and isoform switching during cell differentiation. *Nat. Biotechnol.* **28**, 511–515 (2010).
17. Wei, M., Zhang, R., Zhang, F. & Zhang, Y. Evaluating cell viability heterogeneity based on information fusion of multiple adhesion strengths. *Biotechnol. Bioeng.* **118**, 2360–2367 (2021).
18. Ritz, C., Baty, F., Streibig, J. C. & Gerhard, D. Dose-Response Analysis Using R. *PLoS One* **10**, e0146021 (2015).
19. Yu, J. *et al.* Circular RNA cSMARCA5 inhibits growth and metastasis in hepatocellular carcinoma. *J. Hepatol.* **68**, 1214–1227 (2018).
20. Han, D. *et al.* Circular RNA circMTO1 acts as the sponge of microRNA-9 to suppress hepatocellular carcinoma progression. *Hepatology* **66**, 1151–1164 (2017).
21. Zhong, L. *et al.* Circular RNA circC3P1 suppresses hepatocellular carcinoma growth and metastasis through miR-4641/PCK1 pathway. *Biochem. Biophys. Res. Commun.* **499**, 1044–1049 (2018).
22. Chen, Z. *et al.* circLARP4 induces cellular senescence through regulating miR-761/RUNX3/p53/p21 signaling in hepatocellular carcinoma. *Cancer Sci.* **110**, 568–581 (2019).
23. Qiu, L. *et al.* Circular RNA profiling identifies circADAMTS13 as a miR-484 sponge which suppresses cell proliferation in hepatocellular carcinoma. *Mol. Oncol.* **13**, 441–455 (2019).
24. Chen, L. *et al.* Viral and host inflammation-related factors that can predict the prognosis of hepatocellular carcinoma. *Eur. J. Cancer* **48**, 1977–1987 (2012).
25. Yin, J. *et al.* Effect of antiviral treatment with nucleotide/nucleoside analogs on postoperative prognosis of hepatitis B virus-related hepatocellular carcinoma: a two-stage longitudinal clinical study. *J. Clin. Oncol.* **31**, 3647–3655 (2013).
26. Lukey, M. J. *et al.* Liver-Type Glutaminase GLS2 Is a Druggable Metabolic Node in Luminal-Subtype Breast Cancer. *Cell Rep.* **29**, 76-88.e7 (2019).
27. Kuo, T.-C. *et al.* Glutaminase 2 stabilizes Dicer to repress Snail and metastasis in hepatocellular carcinoma cells. *Cancer Lett.* **383**, 282–294 (2016).
28. Li, Y. *et al.* KLF4-mediated upregulation of CD9 and CD81 suppresses hepatocellular carcinoma development via JNK signaling. *Cell Death Dis.* **11**, 299–312 (2020).
29. Xue, M. *et al.* The association between KLF4 as a tumor suppressor and the prognosis of hepatocellular carcinoma after curative resection. *Aging (Albany NY)* **12**, 15566–15580 (2020).
30. Frühbeck, G. *et al.* The Differential Expression of the Inflammasomes in Adipose Tissue and Colon Influences the Development of Colon Cancer in a Context of Obesity by Regulating Intestinal Inflammation. *J. Inflamm. Res.* **14**, 6431–6446 (2021).
31. Chen, W. *et al.* GTSE1 promotes tumor growth and metastasis by attenuating of KLF4 expression in clear cell renal cell carcinoma. *Lab. Invest.* **102**, 1011–1022 (2022).
32. Ingruber, J. *et al.* KLF4, Slug and EMT in Head and Neck Squamous Cell Carcinoma. *Cells* **10**, 539–560 (2021).
33. Ghaleb, A. M. & Yang, V. W. Krüppel-like factor 4 (KLF4): What we currently know. *Gene* **611**, 27–37 (2017).
34. He, H. *et al.* TRAF7 enhances ubiquitin-degradation of KLF4 to promote hepatocellular carcinoma progression. *Cancer Lett.* **469**, 380–389 (2020).
35. Azar, F. *et al.* Integration of miRNA-regulatory networks in hepatic stellate cells identifies TIMP3 as a key factor in chronic liver disease. *Liver Int.* **40**, 2021–2033 (2020).
36. Liao, Y. *et al.* Dysregulated Sp1/miR-130b-3p/HOXA5 axis contributes to tumor angiogenesis and progression of hepatocellular carcinoma. *Theranostics* **10**, 5209–5224 (2020).
37. Cui, M., Qu, F., Wang, L., Cheng, D. & Liu, X. MiR-18a-5p Facilitates Progression of Hepatocellular Carcinoma by Targeting CPEB3. *Technol. Cancer Res. Treat.* **20**, 15330338211043976 (2021).
38. Guan, L., Ji, D., Liang, N., Li, S. & Sun, B. Up-regulation of miR-10b-3p promotes the progression of hepatocellular carcinoma cells via targeting CMTM5. *J. Cell Mol. Med.* **22**, 3434–3441 (2018).
39. Moshiri, F. *et al.* Circulating miR-106b-3p, miR-101-3p and miR-1246 as diagnostic biomarkers of hepatocellular carcinoma. *Oncotarget* **9**, 15350–15364 (2018).
40. Toh, T. B., Lim, J. J., Hooi, L., Rashid, M. B. M. A. & Chow, E. K.-H. Targeting Jak/Stat pathway as a therapeutic strategy against SP/CD44+ tumorigenic cells in Akt/β-catenin-driven hepatocellular carcinoma. *J. Hepatol.* **72**, 104–118 (2020).
41. Wu, S. *et al.* The pathological significance of LOXL2 in pre-metastatic niche formation of HCC and its related molecular mechanism. *Eur. J. Cancer* **147**, 63–73 (2021).
42. Yang, B. *et al.* High-metastatic cancer cells derived exosomal miR92a-3p promotes epithelial-mesenchymal transition and metastasis of low-metastatic cancer cells by regulating PTEN/Akt pathway in hepatocellular carcinoma. *Oncogene* **39**, 6529–6543 (2020).
43. Liu, C.-X. & Chen, L.-L. Circular RNAs: Characterization, cellular roles, and applications. *Cell* **185**, 2016–2034 (2022).
44. Kristensen, L. S., Jakobsen, T., Hager, H. & Kjems, J. The emerging roles of circRNAs in cancer and oncology. *Nat. Rev. Clin. Oncol.* **19**, 188–206 (2022).
45. He, A. T., Liu, J., Li, F. & Yang, B. B. Targeting circular RNAs as a therapeutic approach: current strategies and challenges. *Signal Transduct. Target. Ther.* **6**, 185–198 (2021).

Figure. S1.

**
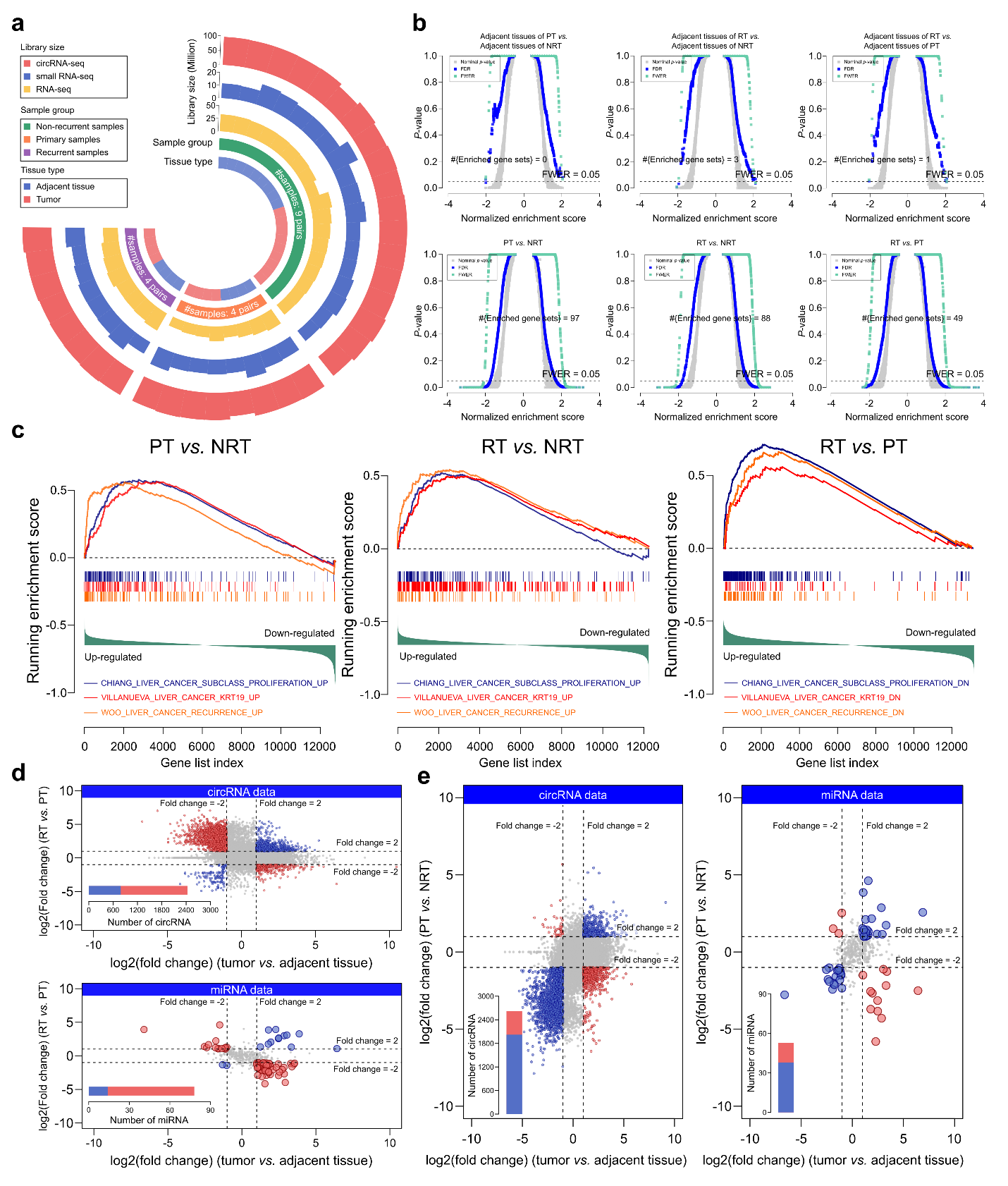
**

**Figure. S1. A multi-omics analysis of mRNA, circRNA, and miRNA expression profiles to characterize the evolution of HCC with the recurrence**. **a** The summary of the circRNA, transcriptome, and small RNA sequencing. **b** The gene sets enriched for the comparisons of three categories of adjacent tissues and tumors: (Top Left) adjacent tissues of primary tumors (PT) vs. those of non-recurrent tumors (NRT); (Top Middle) adjacent tissues of recurrent tumors (RT) vs. those of NRT; and (Top Right) adjacent tissues of RT vs. those of PT. (Bottom Left) PT vs. NRT; (Bottom Middle) RT vs. NRT; and (Bottom Right) RT vs. PT. **c** GSEA plots to characterize three kinds of tumor tissues: (Left) primary tumors (PT) *vs.* non-recurrent tumors (NRT); (Middle) recurrent tumors (RT) *vs.* NRT; and (Right) RT *vs.* PT. **d** Scatter plot of circRNA (top) and miRNA (bottom) data. Fold changes (FCs) between all tumors and paired adjacent tissues are plotted on the x-axis and those between RTs and PTs are on the y-axis. circRNAs (or miRNAs) with FC ≥ 2 are marked with blue if they were up- or downregulated both in the tumors and in the RTs or with red if otherwise. The numbers of red and blue dots are plotted as the stacked bars at the bottom left corner. **e** Scatter plot of circRNA (left) and miRNA (right) data. Fold changes (FCs) between all tumors and paired adjacent tissues were plotted on the X axis and those between PTs and NRTs were on the Y axis. The rest was the same as (**d**).

Figure. S2.


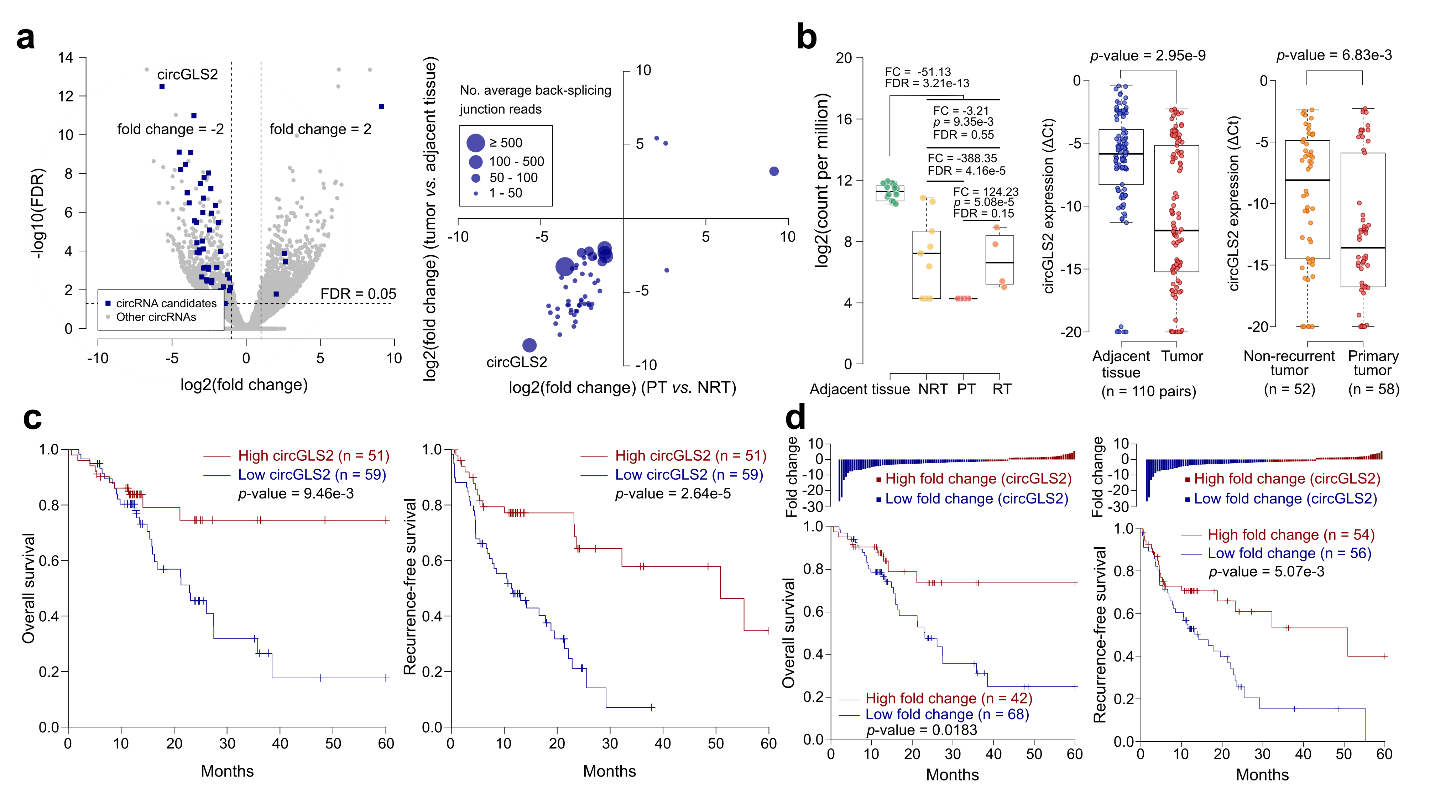


**Figure. S2. Identification of circGLS2 and its association with HCC recurrence. a** Screening of the HCC recurrence-associated circRNAs (Left). Volcano plot for the differential test of tumor *vs.* adjacent tissues. The blue dots represent the circRNA candidates differentially expressed for both tumor *vs.* adjacent tissues and primary tumors *vs.* non-recurrent tumors. Fold changes of the circRNA candidates for the two aforementioned comparisons plotted against the x and y axes (Right). The numbers of average back-splicing junction reads are represented as the sizes of the dots. **b** (Left) Differential expression pattern of circGLS2 in our data. circGLS2 was differentially expressed in the tumors compared with the adjacent tissues and in the primary tumors compared with non-recurrent tumors. (Middle and Right) The PCR-validated differential expression of circGLS2. (Middle) circGLS2 was significantly downregulated in 110 tumors compared with the paired adjacent tissues. (Right) circGLS2 was significantly downregulated in primary tumors compared with non-recurrent tumors. **c** circGLS2 predicted unfavorable prognosis of HCC patients. (Left and Right) The Kaplan–Meier curves for OS (Left) and RFS (Right) differences between two groups of HCC patients with high and low expression levels of circGLS2. **d** circGLS2 was associated with unfavorable overall survival (Left) and recurrence-free survival (Right). The abundance levels of circGLS2 in the tumors were adjusted by the paired adjacent tissues. FC, fold change; FDR, false discovery rate; NRT, non-recurrent tumor; PT, primary tumor; RT, recurrent tumor.

Figure. S3.


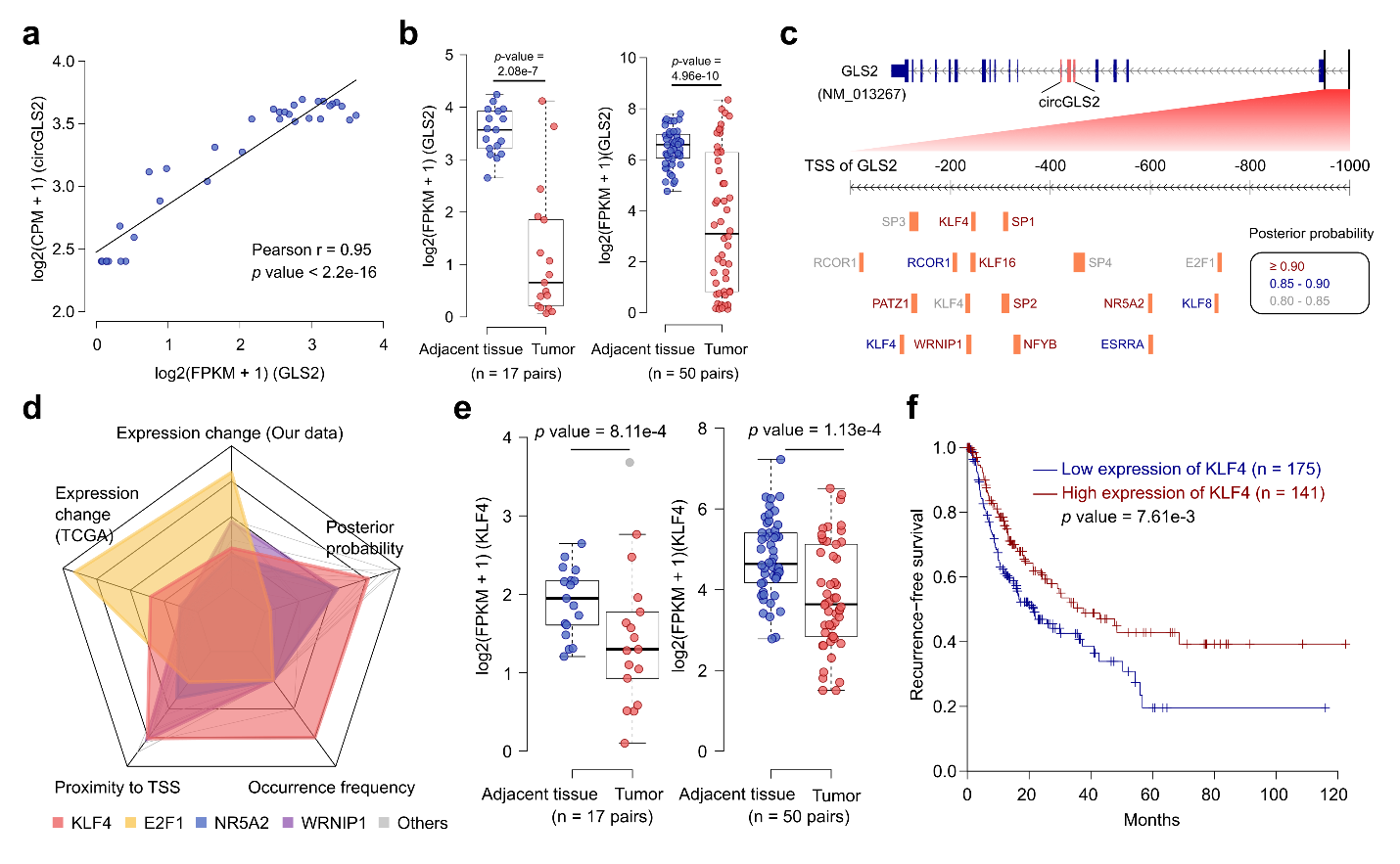


**Figure. S3. Transcriptional factors regulating the expression of GLS2/circGLS2. a** Linear GLS2 was correlated with circGLS2. **b** GLS2 was downregulated in the tumors both in our data (left) and in the TCGA data (right). **c** In total, 17 TFBSs (corresponding to 14 transcription factors) with posterior probabilities >0.8 were found in GLS2’s promoter region. **d** Radar chart of 14 transcription factors. Five aspects were considered: (1) expression changes in our data measured by −log10(FDR); (2) expression changes in the TCGA data measured by −log10(FDR); (3) proximity to GLS2’s TSS; (4) occurrence frequency of the transcription factors; and (5) posterior probabilities assigned by MotEvo. **e** KLF4 was significantly downregulated in the tumor both in our data and in the TCGA data. Note that the outlier marked by the grey dot was removed in our data. **f** Kaplan–Meier plot of recurrence-free survival for two groups of HCC patients with low and high KLF4 expression levels in the TCGA data. FDR, false discovery rate; TFBS, transcription factor binding site; TSS, transcription start site.

Figure. S4.

**
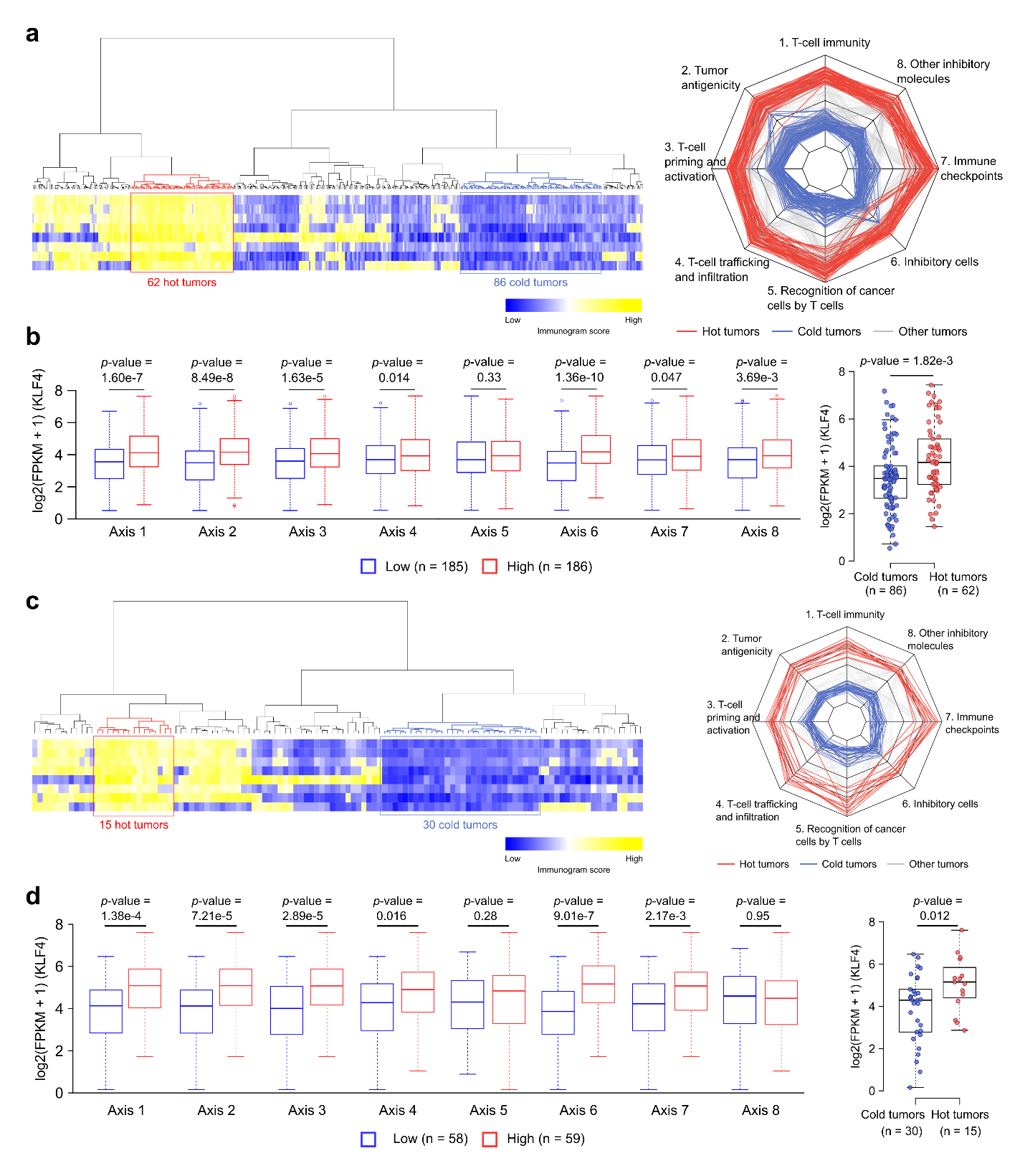
Figure. S4. KLF4 levels were associated with immunological status. a** (Left) Immunogram heatmap of 371 tumors in the TCGA data. The tumors were plotted along columns and eight axes were on the rows. (Right) Eight-axis Immunograms of 371 tumors in the TCGA data. **b** (Left) Boxplots of KLF4 between two groups of tumors with high and low immunogram scores. (Right) The expression levels of KLF4 in the immunologically 86 cold and 62 hot tumors. **c** The heatmap (Left) and radar plot (Right) of immunograms of 117 tumors in five collected transcriptome data sets. **d** (Left) Boxplots of KLF4 between two groups of 117 tumors with high and low immunogram scores. (Right) The expression levels of KLF4 in the immunologically 30 cold and 15 hot tumors.

Figure. S5.

**
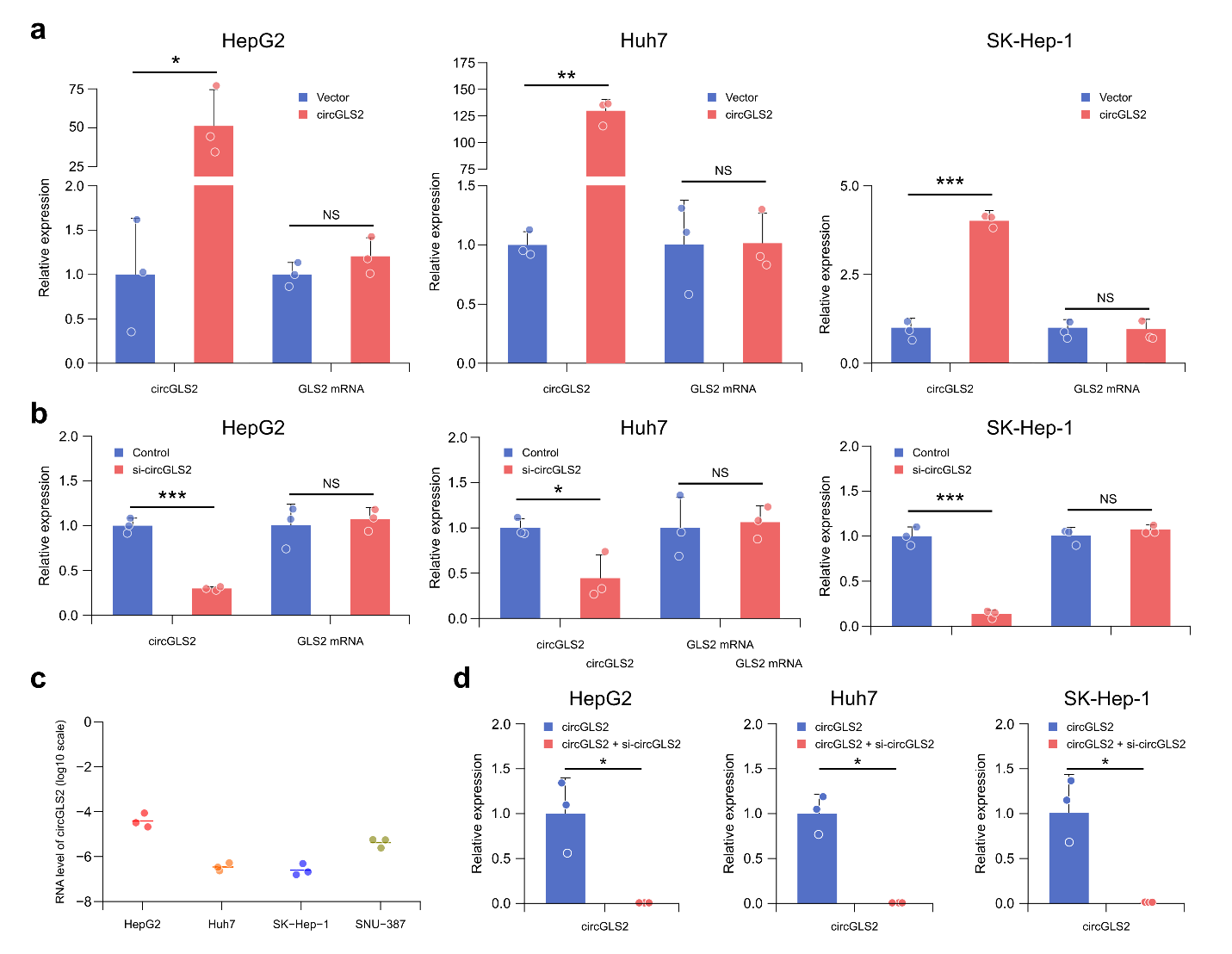
**

**Figure. S5. The overexpression and knockdown effect of circGLS2.** The circGLS2 was overexpressed (**a**) or knocked down (**b**) in HepG2, Huh7, and SK-Hep-1 cell line. **c** The endogenous expression levels in four HCC cell lines. The expression levels were adjusted by those of GAPDH. **d** circGLS2 expression levels in the cells overexpressing circGLS2 compared with those in the cells of circGLS2 + si-circGLS2. The “si-circGLS2” was the mixture of siRNAs; *: *P* value < 0.05; **: *P* value < 0.01; ***: *P* value < 1 × 10 ^-3^.

Figure. S6.


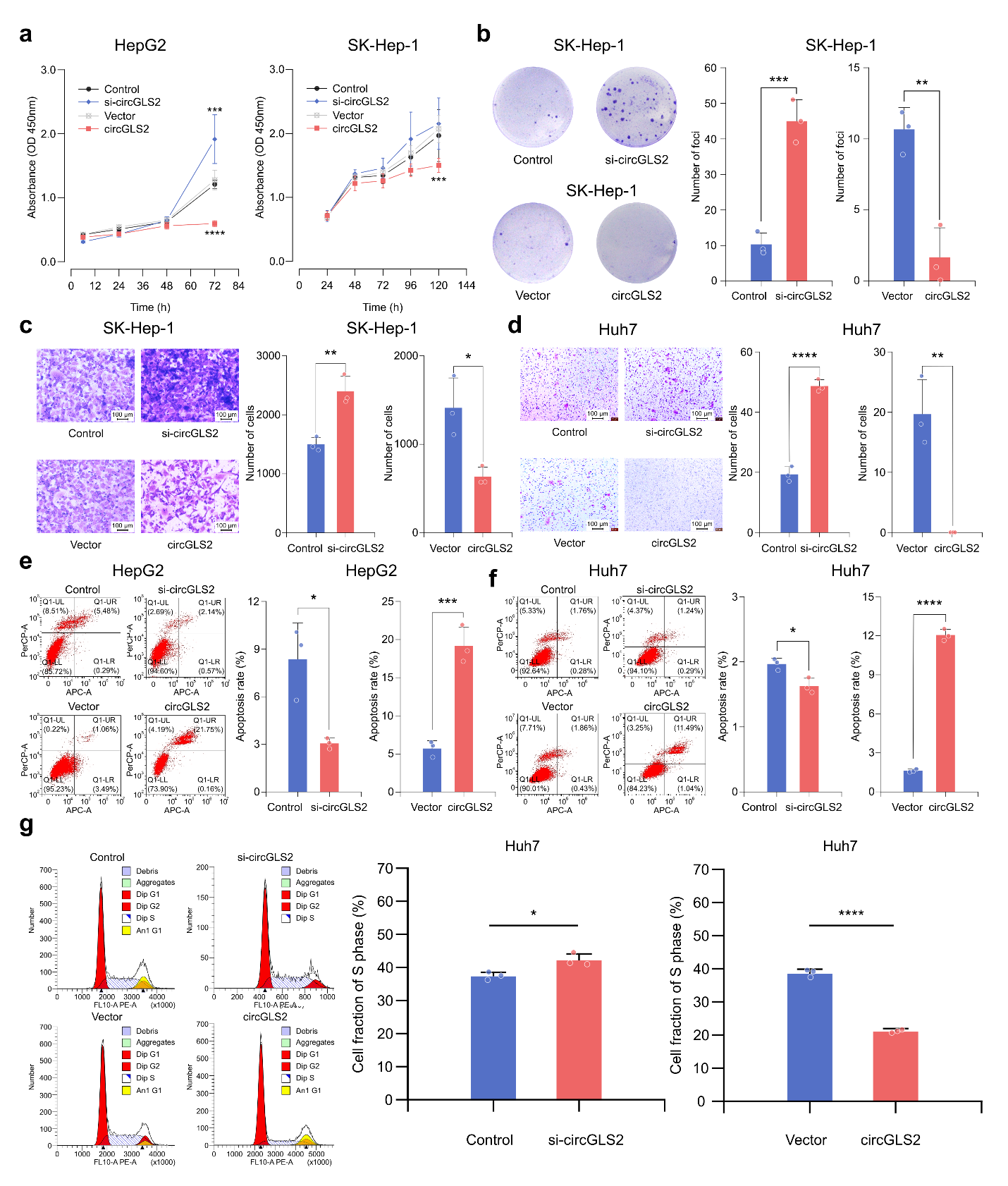


**Figure. S6. Tumor suppressive characteristics of circGLS2 *in vitro*. a** Cell proliferation ability of HepG2 and SK-Hep-1 with circGLS2 overexpressed or knocked down. **b** Colony formation ability of SK-Hep-1 cells with circGLS2 overexpressed or knocked down. **c**, **d** Cell migration ability of SK-Hep-1 and Huh7 cells transfected with siRNAs or overexpression plasmid of circGLS2. **e, f** Apoptosis rate alteration in HepG2 and Huh7 cells transfected with siRNAs or overexpression plasmid of circGLS2. **g** The result of cell cycle assay in the Huh7 with circGLS2 overexpressed or knocked down. The “si-circGLS2” was the mixture of siRNAs. *: *P* value < 0.05; **: *P* value < 0.01; ***: *P* value < 1 × 10 ^-3^; ****: *P* value < 1 × 10 ^-4^.

Figure. S7.


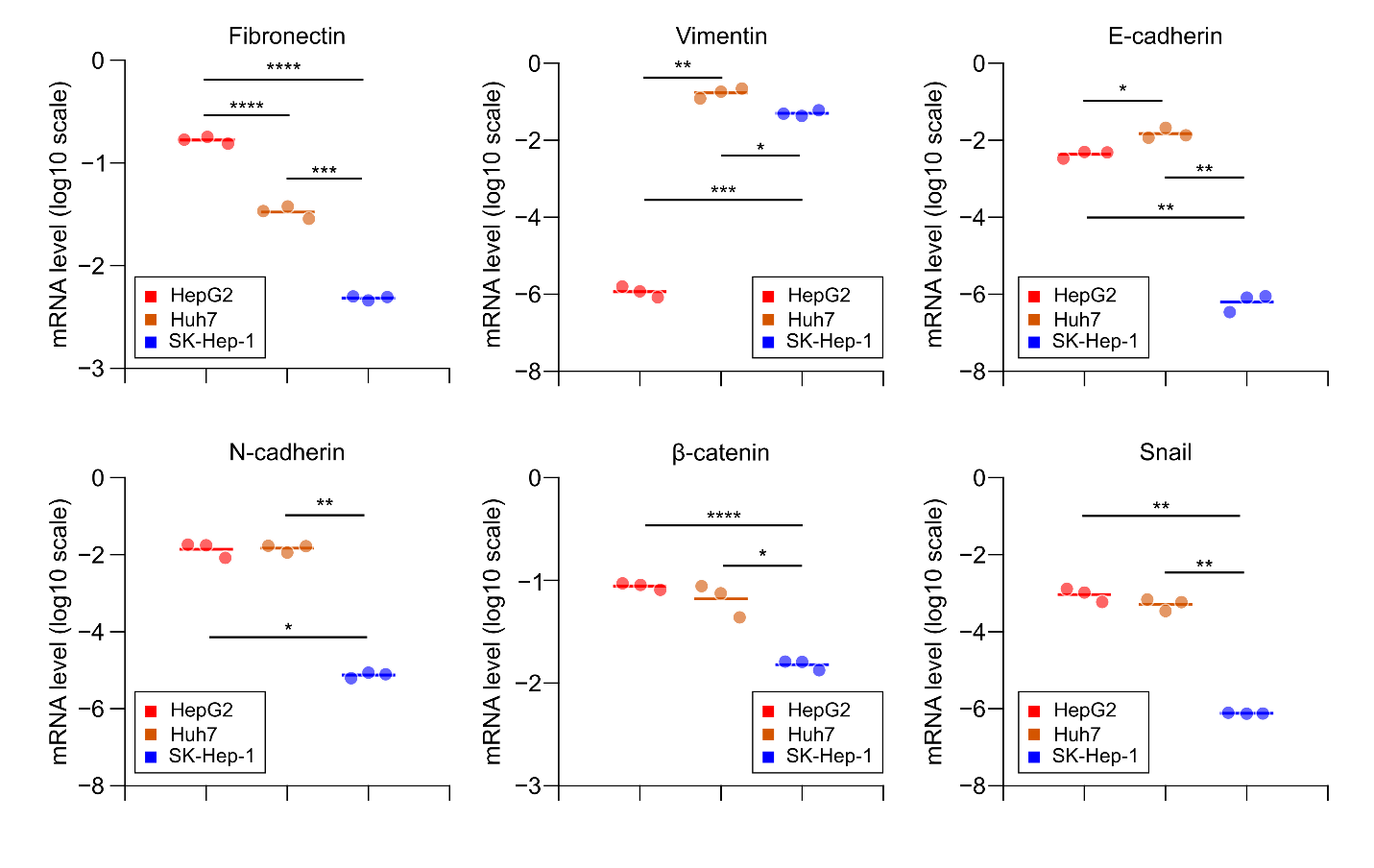


**Figure. S7. The endogenous expression levels of EMT markers in three HCC cell lines.** The expression levels were adjusted by those of GAPDH. *: *P* value < 0.05; **: *P* value < 0.01; ***: *P* value < 1 × 10 ^-3^; ****: *P* value < 1 × 10 ^-4^.

Figure. S8.

**
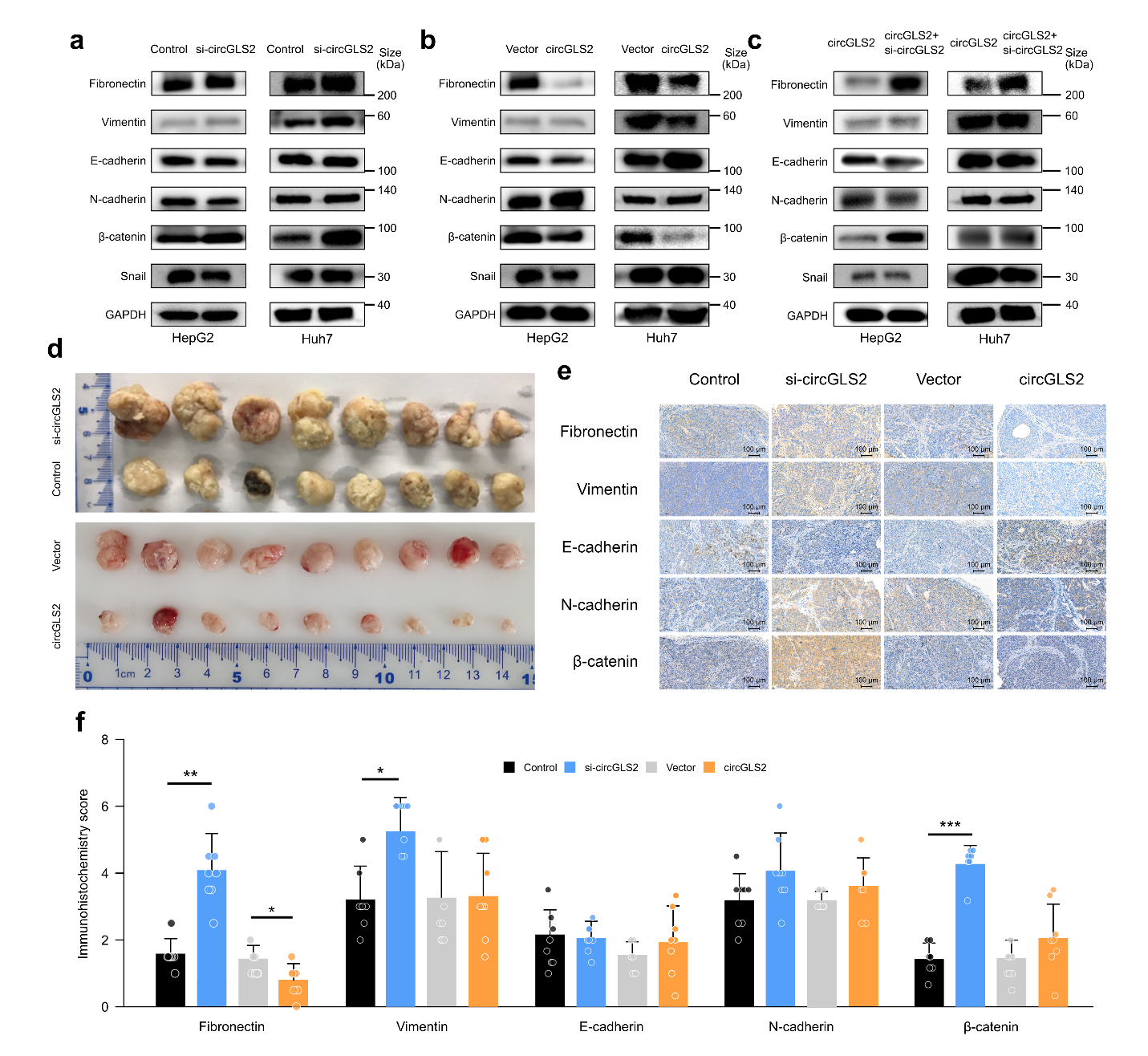
**

**Figure. S8. circGLS2 partially attenuated epithelial–mesenchymal transition (EMT) and its tumor suppressive characteristics *in vivo*.** **a, b, c** The abundance levels of six epithelial–mesenchymal transition markers estimated by Western blot assay in HepG2 and Huh7 transfected with siRNAs or overexpression plasmid of circGLS2. **d** The weight and volume changes of tumors collected from mice injected with circGLS2 siRNA- and overexpression plasmid-transfected Huh7. **e, f** *In vivo* EMT markers in tumor tissues stained by immunohistochemistry. The “si-circGLS2” was the mixture of siRNAs; *: *P* value < 0.05; **: *P* value < 0.01; ***: *P* value < 1 × 10 ^-3^.

Figure. S9.

**
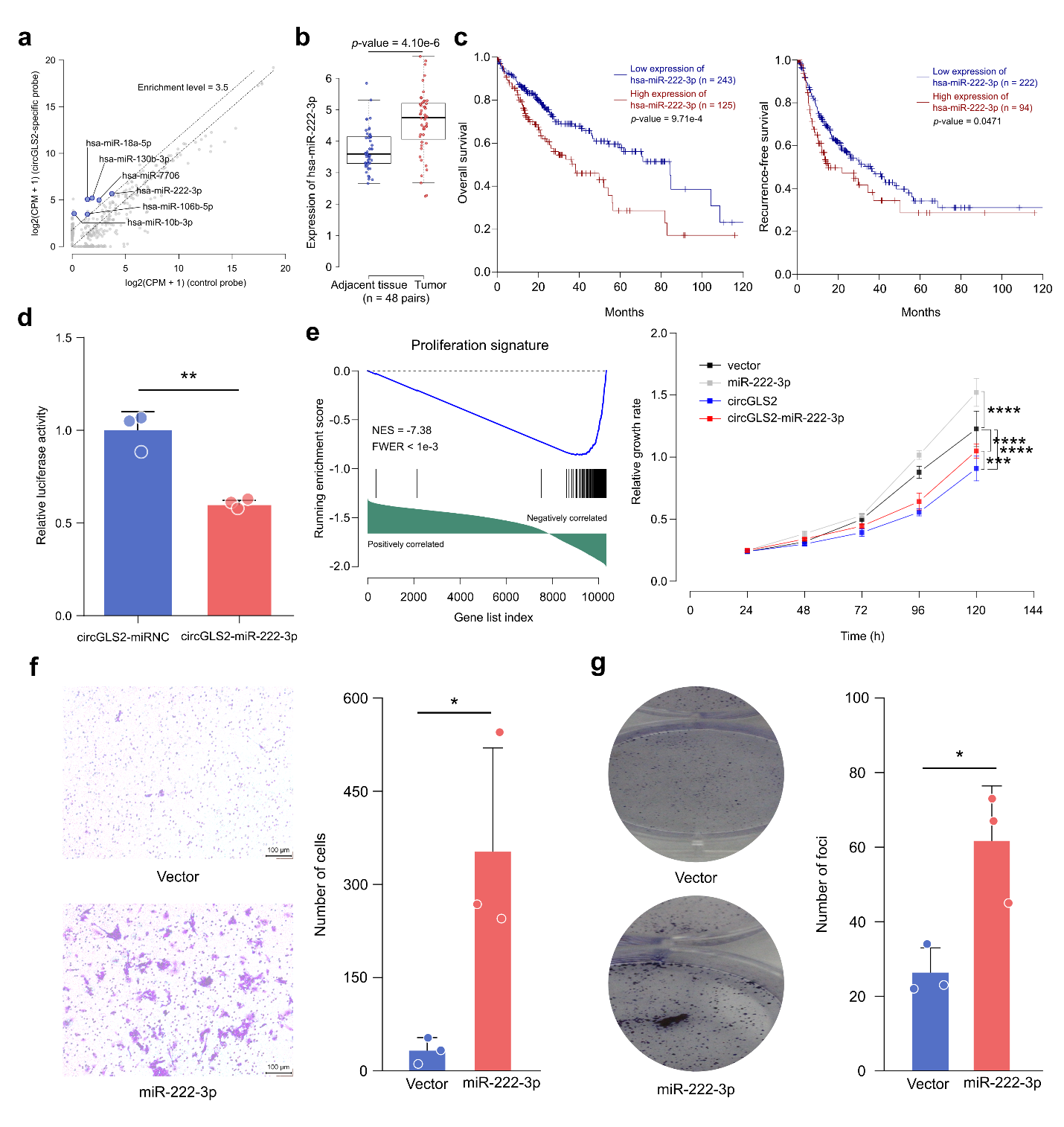
**

**Figure. S9. circGLS2 is a sponge for hsa-miR-222-3p.** **a** Six circGLS2-binding miRNAs obtained by RNA pulldown assay. **b** Box plot of hsa-miR-222-3p expression levels in 48 tumors and paired adjacent tissues of TCGA database. **c** (Left and Right) Kaplan–Meier curves of overall survival and recurrence-free survival for two groups of HCC patients with low and high hsa-miR-222-3p expression levels. Data visualized in Kaplan–Meier curves were retrieved from TCGA datasets. **d** Luciferase reporter assay was performed, and the relative luciferase activity was significantly decreased in the 293T cells co-transfected with overexpression plasmids of circGLS2 and hsa-miR-222-3p. **e** (Left) Enrichment plot of proliferation signature enriched by hsa-miR-222-3p. (Right) Proliferation ability of the HepG2 cells overexpressing hsa-miR-222-3p, circGLS2, or both. **f** and **g** miR-222-3p promoted the cell migration (f) and colony formation (g) ability of HepG2 cells overexpressing miR-222-3p. *: *P* value < 0.05; **: *P* value < 0.01; ***: *P* value < 1 × 10 ^-3^; ****: *P* value < 1 × 10 ^-4^.

Figure. S10.

**
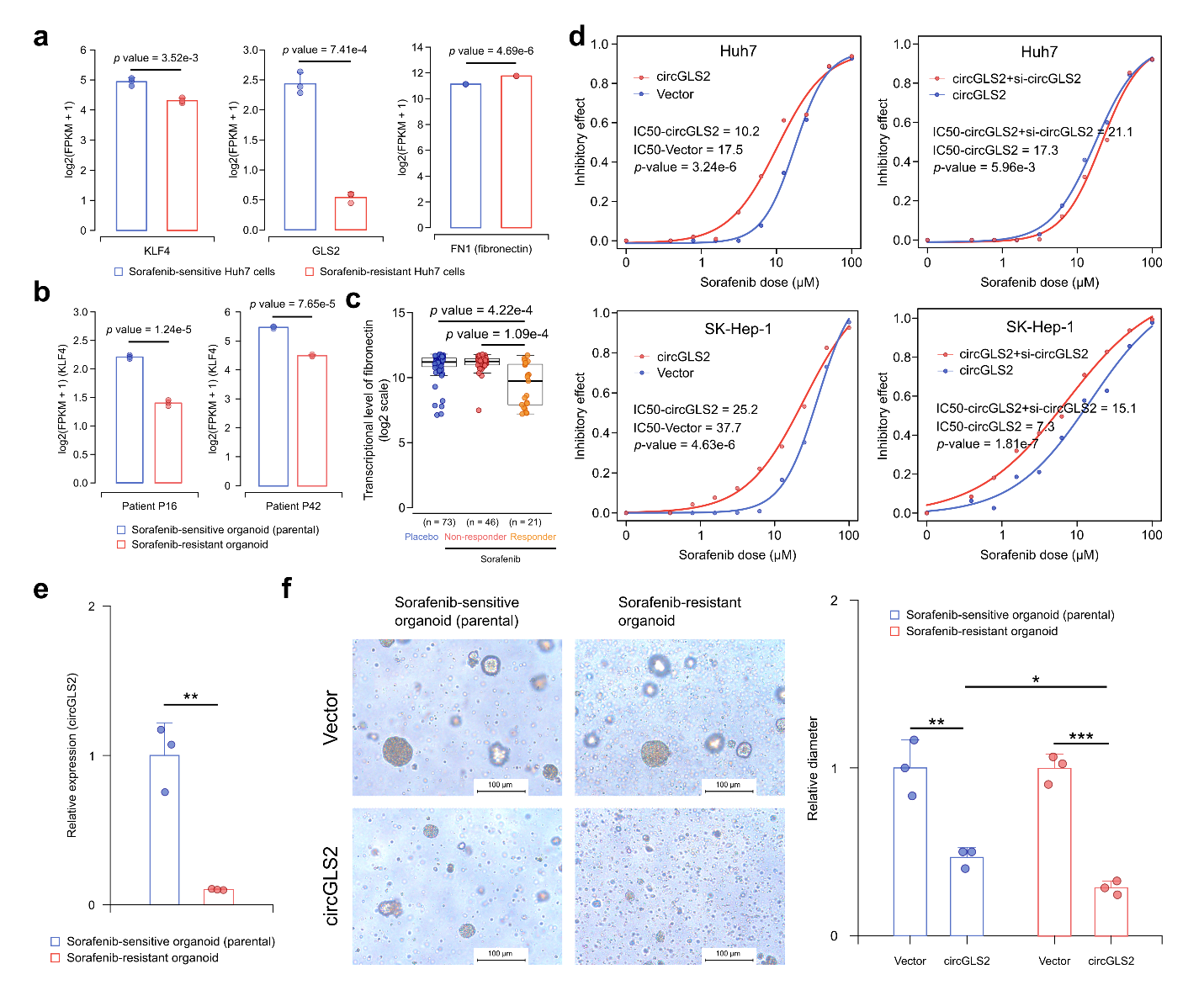
**

**Figure. S10. circGLS2 enhanced sorafenib sensitivity of tumor cells. a** The expression changes of KLF4, GLS2, and FN1 between sorafenib-sensitive and –resistant Huh7 cells. **b** The expression changes of KLF4 in two of our HCC organoids. **c** The expression change of fibronectin in the BIOSTORM microarray data. **d** The IC_50_ assay for sorafenib upon circGLS2 knockdown or overexpression in Huh7 and SK-Hep-1 cells. **e** circGLS2 level was significantly lower in the sorafenib-resistant organoid. **f** circGLS2 was overexpressed in the parental and sorafenib-resistant organoid and their growing ability was compared with each other. The “si-circGLS2” was the mixture of siRNAs. *: *P* value < 0.05; **: *P* value < 0.01; ***: *P* value < 1 × 10 ^-3^.

**Table S1. Baseline characteristics of 13 HCC patients**

| **Variable** |  | **Patients with non-recurrent HCC (n=9)** | **Patients with recurrent HCC (n=4)** |
| --- | --- | --- | --- |
| Age - yr |  | 55.44±10.98 | 52.25±8.22 |
| Gender | Male | 7 (77.8) | 3 (75.0) |
|  | Female | 2 (22.2) | 1 (25.0) |
| Ascites | No | 7 (77.8) | 4 (100.0) |
|  | Yes | 2 (22.2) | 0 (0) |
| Portal vein tumor thrombi | No | 9 (100.0) | 4 (100.0) |
|  | Yes | 0 (0) | 0 (0) |
| Tumor number | Single | 8 (88.9) | 4 (100.0) |
|  | Multiple | 1 (11.1) | 0 (0) |
| Tumor size | <3cm | 3 (33.3) | 0 (0) |
|  | ≥3cm | 6 (66.7) | 4 (100.0) |
| Cirrhosis | No | 4 (44.4) | 3 (75.0) |
|  | Mild cirrhosis | 0 (0) | 0 (0) |
|  | Cirrhosis | 5 (55.6) | 1 (25.0) |
| Microsatellite | No | 9 (100.0) | 3 (75.0) |
|  | Yes | 0 (0) | 1 (25.0) |
| Microscopic vascular invasion | No | 7 (77.8) | 3 (75.0) |
|  | Yes | 2 (22.2) | 1 (25.0) |
| Tumor differentiation | I | 0 (0) | 0 (0) |
|  | II | 7 (77.8) | 1 (25.0) |
|  | III | 2 (22.2) | 3 (75.0) |
| BCLC stage | 0 | 1 (11.1) | 0 (0) |
|  | A | 7 (77.8) | 4 (100) |
|  | B | 1 (11.1) | 0 (0) |
|  | C | 0 (0) | 0 (0) |
| HBeAg | Negative | 0 (0) | 0 (0) |
|  | Positive | 9 (100) | 4 (100) |
| Total bilirubin (umol/L) | ≤20 | 7 (77.8) | 4 (100) |
|  | >20 | 2 (22.2) | 0 (0) |
| Direct bilirubin (umol/L) | ≤7 | 8 (88.9) | 4 (100) |
|  | >7 | 1 (11.1) | 0 (0) |
| Albumin (g/L) | 35-55 | 8 (88.9) | 4 (100) |
|  | <35 OR >55 | 1 (11.1) | 0 (0) |
| Alpha-fetoprotein (ng/ml) | ≤20 | 2 (22.2) | 1 (25.0) |
|  | >20 | 7 (77.8) | 3 (75.0) |
| Follow-up time (month) | Median | 75 | 66 |
|  | IQR | 67.0-76.0 | 51.75-70.75 |
| HCC-related death | No | 3 (33.3) | 1 (25.0) |
|  | Yes | 6 (66.7) | 3 (75.0) |
| Recurrence after surgery (month) | Median | NA | 21.5 |
|  | IQR | NA | 18.5-23.0 |

† Plus-minus values are means ± SD; Data are number (%), unless otherwise indicated. AST, aspartate aminotransferase. AFP, alpha-fetoprotein. ALT, alanine aminotransferase. HCC, hepatocellular carcinoma.

| **Table S2. Baseline characteristics of 110 HCC patients†** | | |
| --- | --- | --- |
| **Variable** |  | **Patients with non-recurrent HCC (n=110)** |
| Age - yr |  | 55.25±11.96 |
| Gender | Male | 93 (84.55) |
|  | Female | 17 (15.45) |
| Ascites | No | 110 (100) |
|  | Yes | 0 (0) |
| Portal vein tumor thrombi | No | 110 (100) |
|  | Yes | 0 (0) |
| Tumor number | Single | 87 (79.09) |
|  | Multiple | 23 (20.91) |
| Tumor size | <3cm | 1 (0.91) |
|  | ≥3cm | 109 (99.09) |
| Cirrhosis | No | 64 (58.18) |
|  | Mild cirrhosis | 37 (33.64) |
|  | Cirrhosis | 9 (8.18) |
| Microsatellite | No | 87 (79.09) |
|  | Yes | 23 (20.91) |
| Microscopic vascular invasion | No | 75 (68.18) |
|  | Yes | 35 (31.82) |
| Tumor differentiation | I | 1 (0.91) |
|  | II | 25 (22.73) |
|  | III | 84 (76.36) |
| BCLC stage | 0 | 1 (0.91) |
|  | A | 24 (21.82) |
|  | B | 85 (77.27) |
| HBeAg | Negative | 85 (77.27) |
|  | Positive | 25 (22.73) |
| Total bilirubin (umol/L) | ≤20 | 96 (87.27) |
|  | >20 | 14 (12.73) |
| Direct bilirubin (umol/L) | ≤7 | 84 (76.36) |
|  | >7 | 26 (23.64) |
| Albumin (g/L) | 35-55 | 102 (92.73) |
|  | <35 OR >55 | 8 (7.27) |
| Alpha-fetoprotein (ng/ml) | ≤20 | 49 (44.55) |
|  | >20 | 61 (55.45) |
| Follow-up time (month) | Median | 13.1 |
|  | IQR | 11.4-25.4 |
| HCC-related death | No | 71 (64.55) |
|  | Yes | 39 (35.45) |
| Recurrence | No | 52 (47.3) |
|  | Yes | 58 (52.7) |
| Recurrence after surgery (month) | Median | 11.9 |
|  | IQR | 3.5-16.0 |

† Plus-minus values are means ± SD; Data are number (%), unless otherwise indicated.

Abbreviation: AFP, alpha-fetoprotein. HCC, hepatocellular carcinoma.

**Table S5. The univariable Cox analysis of 110 HCC patients’ recurrence**

| **Variable** |  | **Hazard ratio (95% CI)** | ***P* value** |
| --- | --- | --- | --- |
| Age - yr |  | 0.99 (0.97 - 1.02) | 0.67 |
| Gender | Male | Reference |  |
|  | Female | 0.71 (0.33 - 1.50) | 0.35 |
| **Tumor number** | **Single** | **Reference** |  |
|  | **Multiple** | **1.46 (1.13 - 1.89)** | **0.01** |
| Cirrhosis | No | Reference |  |
|  | Mild | 0.83 (0.47 - 1.47) | 0.53 |
|  | Yes | 0.79 (0.31 - 2.03) | 0.62 |
| **Microscopic vascular invasion** | **No** | **Reference** |  |
|  | **Yes** | **1.83 (1.08 - 3.10)** | **0.03** |
| **Microsatellite** | **No** | **Reference** |  |
|  | **Yes** | **1.88 (1.05 - 3.36)** | **0.04** |
| BCLC stage | 0/A | Reference |  |
|  | B | 1.27 (0.67 - 2.41) | 0.46 |
| Alpha-fetoprotein (ng/ml) | ≤20 | Reference |  |
|  | >20 | 1.58 (0.92 - 2.71) | 0.09 |
| HBeAg | Negative | Reference |  |
|  | Positive | 0.90 (0.48 - 1.66) | 0.72 |
| Total bilirubin (umol/L) | ≤20 | Reference |  |
|  | >20 | 1.06 (0.48 - 2.36) | 0.88 |
| Direct bilirubin (umol/L) | ≤7 | Reference |  |
|  | >7 | 1.28 (0.69 - 2.35) | 0.44 |
| Albumin (g/L) | 35-55 | Reference |  |
|  | <35 OR >55 | 1.03 (0.41 - 2.59) | 0.95 |
| **circGLS2** | **ΔCt < -10.93** | **Reference** |  |
|  | **ΔCt ≥ -10.93** | **0.29 (0.16 - 0.54)** | **2.47E-5** |

Abbreviation: CI, confidence interval; HCC, hepatocellular carcinoma.

**Table S6.** **miRNA candidates binding circGLS2 obtained by miRNA pulldown assay**

| **miRNA** | **miRNA pulldown assay** | | | **tumor *vs.* adjacent tissue** | | |
| --- | --- | --- | --- | --- | --- | --- |
|  | **CPM (control probe)** | **CPM (cirGLS2-specific probe)** | **Enrichment level** | **log2(FC)** | **p value** | **FDR** |
| hsa-miR-222-3p | 12.15 | 49.95 | 3.86 | 1.08 | 3.52E-03 | 1.60E-02 |
| hsa-miR-130b-3p | 2.65 | 35.94 | 10.12 | 1.43 | 7.96E-04 | 5.56E-03 |
| hsa-miR-18a-5p | 1.68 | 32.43 | 12.48 | 2.84 | 4.47E-07 | 4.28E-05 |
| hsa-miR-7706 | 4.723 | 30.61 | 5.53 | 1.18 | 2.43E-03 | 1.30E-02 |
| hsa-miR-10b-3p | 0.13 | 10.58 | 10.25 | 1.96 | 3.56E-03 | 1.60E-02 |
| hsa-miR-106b-5p | 1.68 | 9.95 | 4.09 | 1.15 | 1.96E-03 | 1.10E-02 |

Abbreviation: CPM, count per million; FDR, false discovery rate.

**Table S7. Thirty-three common gene sets negatively and positively enriched by 5 miRNAs and circGLS2***

| **Gene set** | **Normalized enrichment score** | | | | |
| --- | --- | --- | --- | --- | --- |
|  | **miR-222-3p** | **miR-130b-3p** | **miR-18a-5p** | **miR-10b-3p** | **miR-106b-5p** |
| CAIRO_HEPATOBLASTOMA_DN | -7.49 | -6.01 | -6.20 | -6.89 | -6.77 |
| CHIANG_LIVER_CANCER_SUBCLASS_PROLIFERATION_DN | -7.38 | -6.51 | -6.50 | -6.77 | -7.01 |
| HSIAO_LIVER_SPECIFIC_GENES | -6.75 | -6.12 | -5.95 | -6.02 | -6.27 |
| HOSHIDA_LIVER_CANCER_SUBCLASS_S3 | -6.73 | -6.03 | -5.98 | -6.16 | -6.47 |
| LEE_LIVER_CANCER_SURVIVAL_UP | -6.35 | -5.79 | -5.37 | -5.33 | -5.76 |
| OHGUCHI_LIVER_HNF4A_TARGETS_DN | -5.91 | -5.24 | -5.14 | -4.94 | -5.44 |
| WOO_LIVER_CANCER_RECURRENCE_DN | -5.80 | -5.38 | -5.06 | -5.13 | -5.44 |
| BOYAULT_LIVER_CANCER_SUBCLASS_G123_DN | -5.76 | -5.13 | -5.06 | -5.58 | -5.23 |
| ANDERSEN_LIVER_CANCER_KRT19_DN | -5.31 | -4.94 | -5.08 | -4.98 | -5.36 |
| VILLANUEVA_LIVER_CANCER_KRT19_DN | -5.23 | -4.64 | -4.76 | -4.46 | -4.86 |
| YAMASHITA_LIVER_CANCER_STEM_CELL_DN | -5.13 | -4.59 | -4.53 | -4.43 | -4.78 |
| LEE_LIVER_CANCER_DENA_DN | -5.12 | -4.76 | -4.38 | -4.65 | -4.89 |
| SU_LIVER | -5.03 | -4.49 | -4.63 | -4.58 | -4.50 |
| BOYAULT_LIVER_CANCER_SUBCLASS_G3_DN | -5.01 | -4.59 | -4.34 | -3.85 | -4.77 |
| LEE_LIVER_CANCER_MYC_TGFA_DN | -4.85 | -4.00 | -3.98 | -3.85 | -4.49 |
| CAIRO_LIVER_DEVELOPMENT_DN | -4.78 | -4.64 | -4.41 | -3.66 | -4.57 |
| LEE_LIVER_CANCER_MYC_E2F1_DN | -4.71 | -4.12 | -3.94 | -3.97 | -4.66 |
| VECCHI_GASTRIC_CANCER_EARLY_DN | -4.59 | -4.58 | -4.35 | -4.23 | -4.60 |
| CAIRO_HEPATOBLASTOMA_CLASSES_DN | -4.58 | -4.92 | -4.79 | -4.75 | -4.76 |
| KIM_LIVER_CANCER_POOR_SURVIVAL_DN | -4.52 | -4.44 | -4.29 | -3.59 | -4.63 |
| LEE_LIVER_CANCER_E2F1_DN | -4.50 | -3.87 | -3.74 | -3.42 | -4.30 |
| SERVITJA_LIVER_HNF1A_TARGETS_DN | -4.46 | -4.13 | -3.94 | -4.18 | -4.49 |
| LEE_LIVER_CANCER_CIPROFIBRATE_DN | -4.45 | -4.04 | -3.72 | -3.85 | -4.16 |
| ACEVEDO_LIVER_TUMOR_VS_NORMAL_ADJACENT_TISSUE_DN | -4.43 | -3.98 | -4.04 | -4.22 | -4.34 |
| NAKAYAMA_SOFT_TISSUE_TUMORS_PCA2_DN | -4.38 | -3.67 | -3.39 | -3.68 | -3.97 |
| CHIANG_LIVER_CANCER_SUBCLASS_POLYSOMY7_UP | -4.36 | -3.64 | -3.90 | -3.28 | -3.97 |
| LEE_LIVER_CANCER_ACOX1_DN | -4.34 | -4.07 | -3.77 | -3.71 | -4.15 |
| SHETH_LIVER_CANCER_VS_TXNIP_LOSS_PAM4 | -4.27 | -4.16 | -3.94 | -3.86 | -4.31 |
| BOYAULT_LIVER_CANCER_SUBCLASS_G1_DN | -4.22 | -3.94 | -4.33 | -3.91 | -4.05 |
| MINGUEZ_LIVER_CANCER_VASCULAR_INVASION_DN | -4.16 | -3.72 | -3.66 | -3.95 | -3.79 |
| MOOTHA_FFA_OXYDATION | -3.76 | -3.52 | -3.31 | -3.10 | -3.55 |
| VARELA_ZMPSTE24_TARGETS_DN | -3.56 | -3.39 | -3.39 | -2.94 | -3.31 |
| DELYS_THYROID_CANCER_DN | -3.56 | -3.90 | -3.40 | -3.96 | -3.94 |

* Familywise error rates of all gene sets were < 1e-3.
