# Supplementary material for: circGLS2 inhibits hepatocellular carcinoma recurrence via regulating hsa-miR-222-3p–PTEN–AKT signaling
Source: Signal Transduct Target Ther. 2023 Feb 17;8:67. doi: 10.1038/s41392-022-01275-6 (PMC9935627; doi:10.1038/s41392-022-01275-6)
Supplement: Supplementary file 2 — Supplementary Figures [file 41392_2022_1275_MOESM2_ESM.pdf]

Figure. S1.

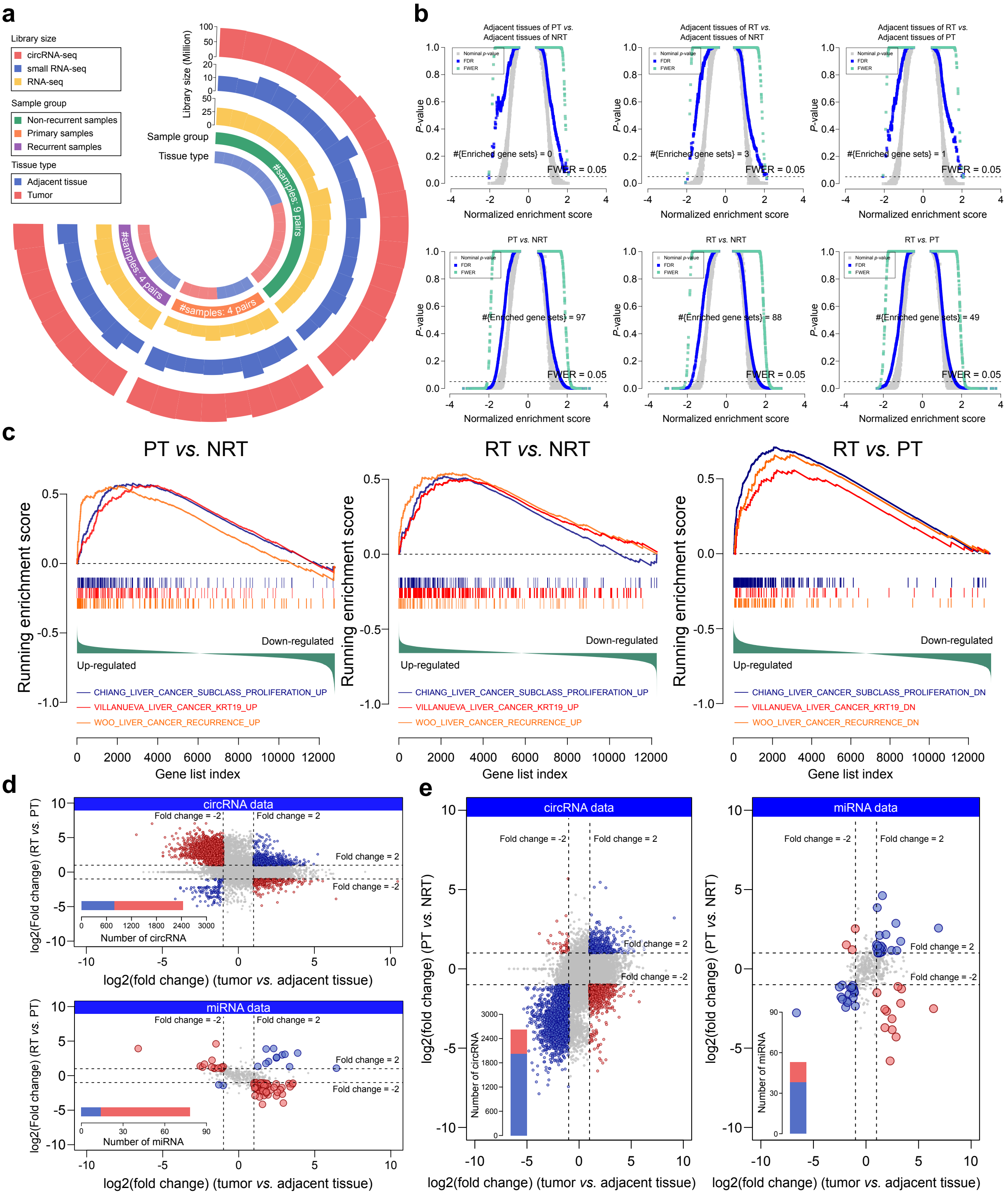

Figure. S1. A multi-omics analysis of mRNA, circRNA, and miRNA expression profiles to characterize the evolution of HCC with the recurrence. **a** The summary of the circRNA, transcriptome, and small RNA sequencing. **b** The gene sets enriched for the comparisons of three categories of adjacent tissues and tumors: (Top Left) adjacent tissues of primary tumors (PT) vs. those of non-recurrent tumors (NRT); (Top Middle) adjacent tissues of recurrent tumors (RT) vs.

those of NRT; and (Top Right) adjacent tissues of RT vs. those of PT. (Bottom Left) PT vs. NRT; (Bottom Middle) RT vs. NRT; and (Bottom Right) RT vs. PT. **c** GSEA plots to characterize three kinds of tumor tissues: (Left) primary tumors (PT) *vs.* non-recurrent tumors (NRT); (Middle) recurrent tumors (RT) *vs.* NRT; and (Right) RT *vs.* PT. **d** Scatter plot of circRNA (top) and miRNA (bottom) data. Fold changes (FCs) between all tumors and paired adjacent tissues are plotted on the x-axis and those between RTs and PTs are on the y-axis. circRNAs (or miRNAs) with FC ≥ 2 are marked with blue if they were up- or downregulated both in the tumors and in the RTs or with red if otherwise. The numbers of red and blue dots are plotted as the stacked bars at the bottom left corner. **e** Scatter plot of circRNA (left) and miRNA (right) data. Fold changes (FCs) between all tumors and paired adjacent tissues were plotted on the X axis and those between PTs and NRTs were on the Y axis. The rest was the same as (**d**).

Figure. S2.

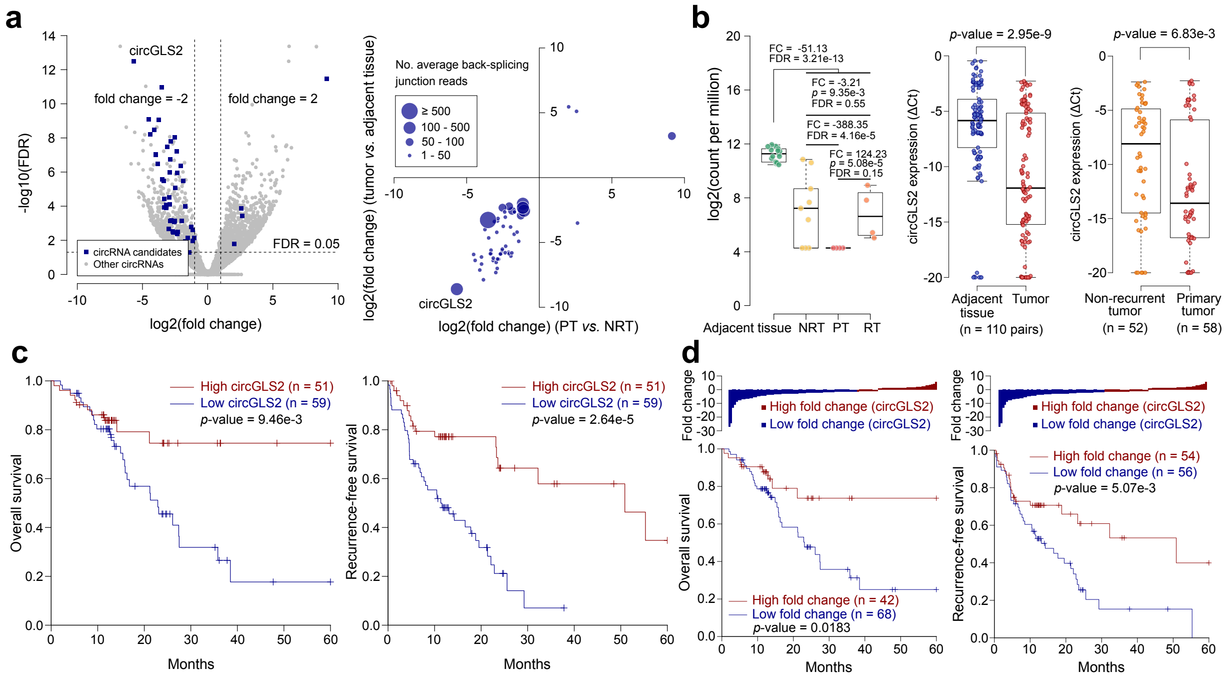

Figure. S2. Identification of circGLS2 and its association with HCC recurrence. a Screening of the HCC recurrence-associated circRNAs (Left). Volcano plot for the differential test of tumor *vs.* adjacent tissues. The blue dots represent the circRNA candidates differentially expressed for both tumor *vs.* adjacent tissues and primary tumors *vs.* non-recurrent tumors. Fold changes of the circRNA candidates for the two aforementioned comparisons plotted against the x and y axes (Right). The numbers of average back-splicing junction reads are represented as the sizes of the dots. **b** (Left) Differential expression pattern of circGLS2 in our data. circGLS2 was differentially expressed in the tumors compared with the adjacent tissues and in the primary tumors compared with non-recurrent tumors. (Middle and Right) The PCR-validated differential expression of circGLS2. (Middle) circGLS2 was significantly downregulated in 110 tumors compared with the paired adjacent tissues. (Right) circGLS2 was significantly downregulated in primary tumors compared with non-recurrent tumors. **c** circGLS2 predicted unfavorable prognosis of HCC patients. (Left and Right) The Kaplan–Meier curves for OS (Left) and RFS (Right) differences between two groups of HCC patients with high and low expression levels of circGLS2. **d** circGLS2 was associated with unfavorable overall survival (Left) and recurrence-free survival (Right). The abundance levels of circGLS2 in the tumors were adjusted by the paired adjacent tissues. FC, fold change; FDR, false discovery rate; NRT, non-recurrent tumor; PT, primary tumor; RT, recurrent tumor.

Figure. S3.

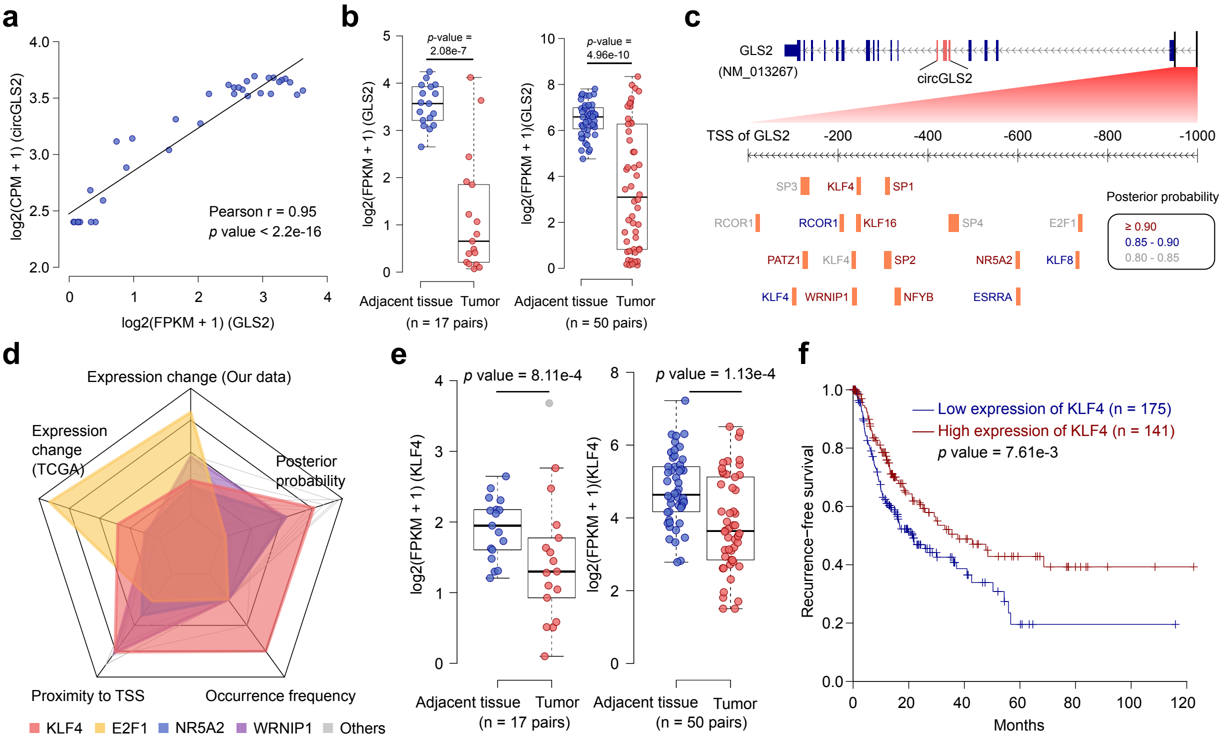

Figure. S3. Transcriptional factors regulating the expression of GLS2/circGLS2. a Linear GLS2 was correlated with circGLS2. **b** GLS2 was downregulated in the tumors both in our data (left) and in the TCGA data (right). **c** In total, 17 TFBSs (corresponding to 14 transcription factors) with posterior probabilities >0.8 were found in GLS2's promoter region. **d** Radar chart of 14 transcription factors. Five aspects were considered: (1) expression changes in our data measured by −log10(FDR); (2) expression changes in the TCGA data measured by −log10(FDR); (3) proximity to GLS2's TSS; (4) occurrence frequency of the transcription factors; and (5) posterior probabilities assigned by MotEvo. **e** KLF4 was significantly downregulated in the tumor both in our data and in the TCGA data. Note that the outlier marked by the grey dot was removed in our data. **f** Kaplan–Meier plot of recurrence-free survival for two groups of HCC patients with low and high KLF4 expression levels in the TCGA data. FDR, false discovery rate; TFBS, transcription factor binding site; TSS, transcription start site.

Figure. S4.

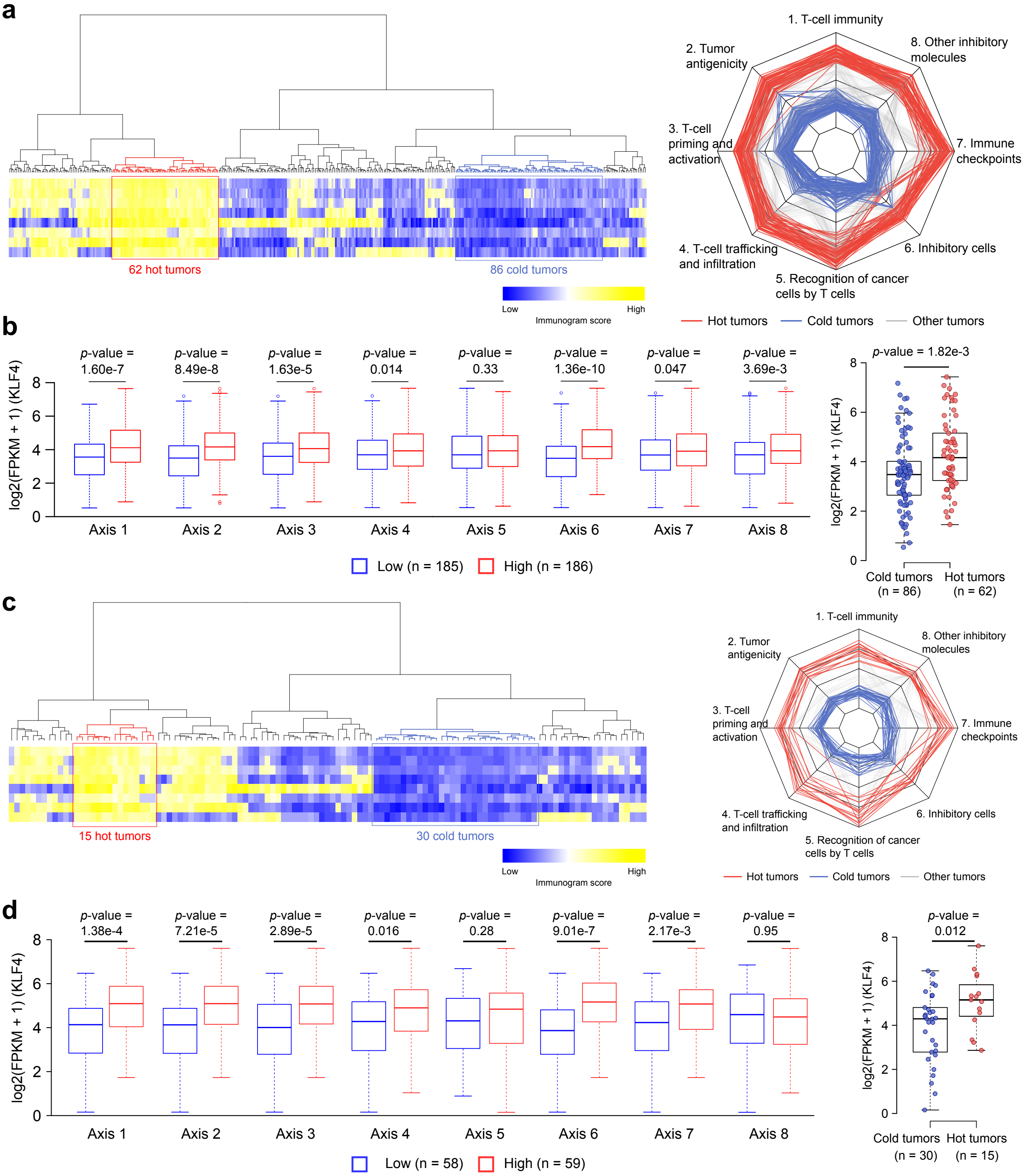

Figure. S4. KLF4 levels were associated with immunological status. a (Left) Immunogram heatmap of 371 tumors in the TCGA data. The tumors were plotted along columns and eight axes were on the rows. (Right) Eight-axis Immunograms of 371 tumors in the TCGA data. **b** (Left) Boxplots of KLF4 between two groups of tumors with high and low immunogram scores. (Right) The expression levels of KLF4 in the immunologically 86 cold and 62 hot tumors. **c** The heatmap (Left) and radar plot (Right) of immunograms of 117 tumors in five collected

transcriptome data sets. **d** (Left) Boxplots of KLF4 between two groups of 117 tumors with high and low immunogram scores. (Right) The expression levels of KLF4 in the immunologically 30 cold and 15 hot tumors.

Figure. S5.

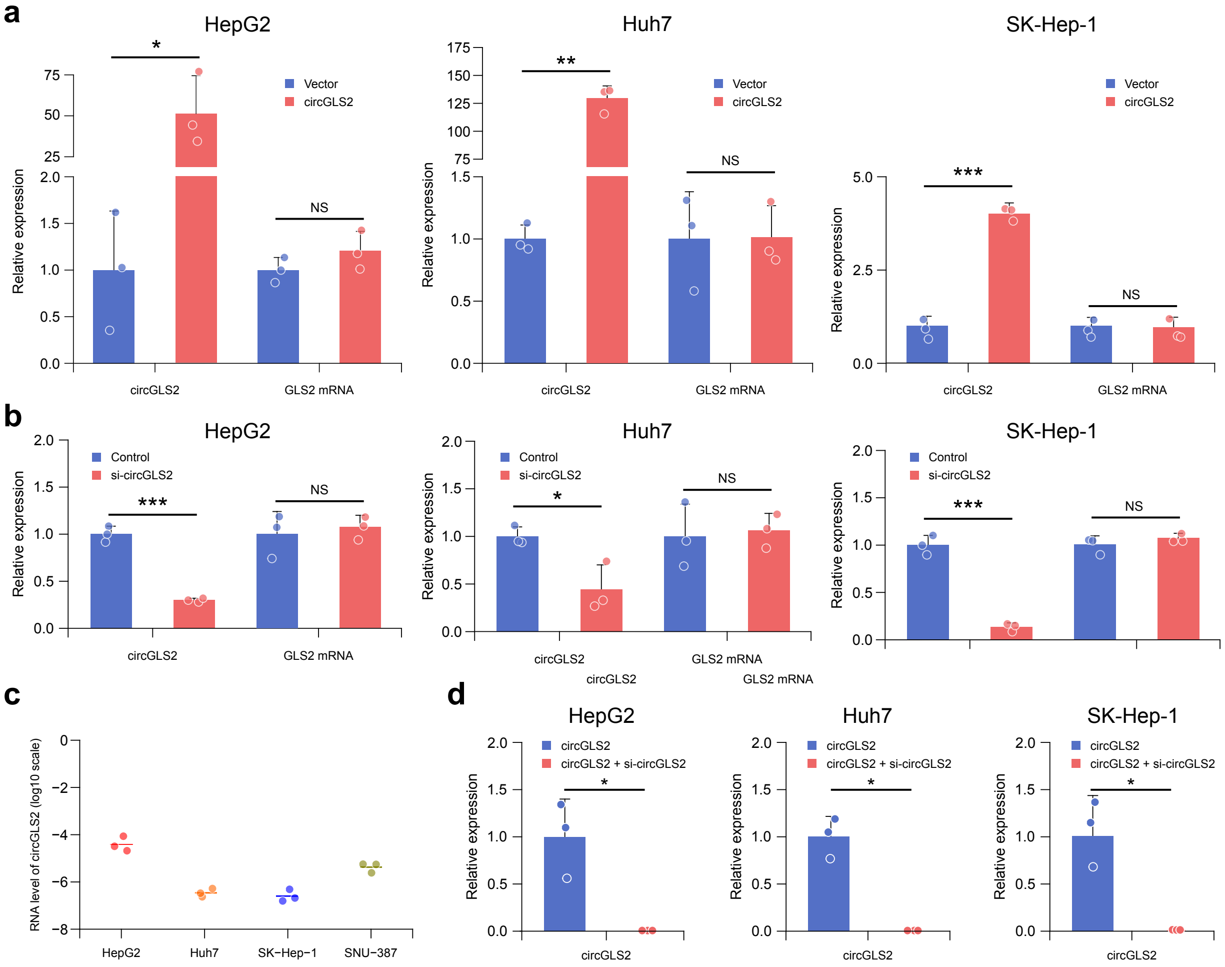

Figure. S5. The overexpression and knockdown effect of circGLS2. The circGLS2 was overexpressed (**a**) or knocked down (**b**) in HepG2, Huh7, and SK-Hep-1 cell line. **c** The endogenous expression levels in four HCC cell lines. The expression levels were adjusted by those of GAPDH. **d** circGLS2 expression levels in the cells overexpressing circGLS2 compared with those in the cells of circGLS2 + si-circGLS2. The “si-circGLS2” was the mixture of siRNAs; *: P value < 0.05; **: P value < 0.01; ***: P value < 1×10^{-3}.

Figure. S6.

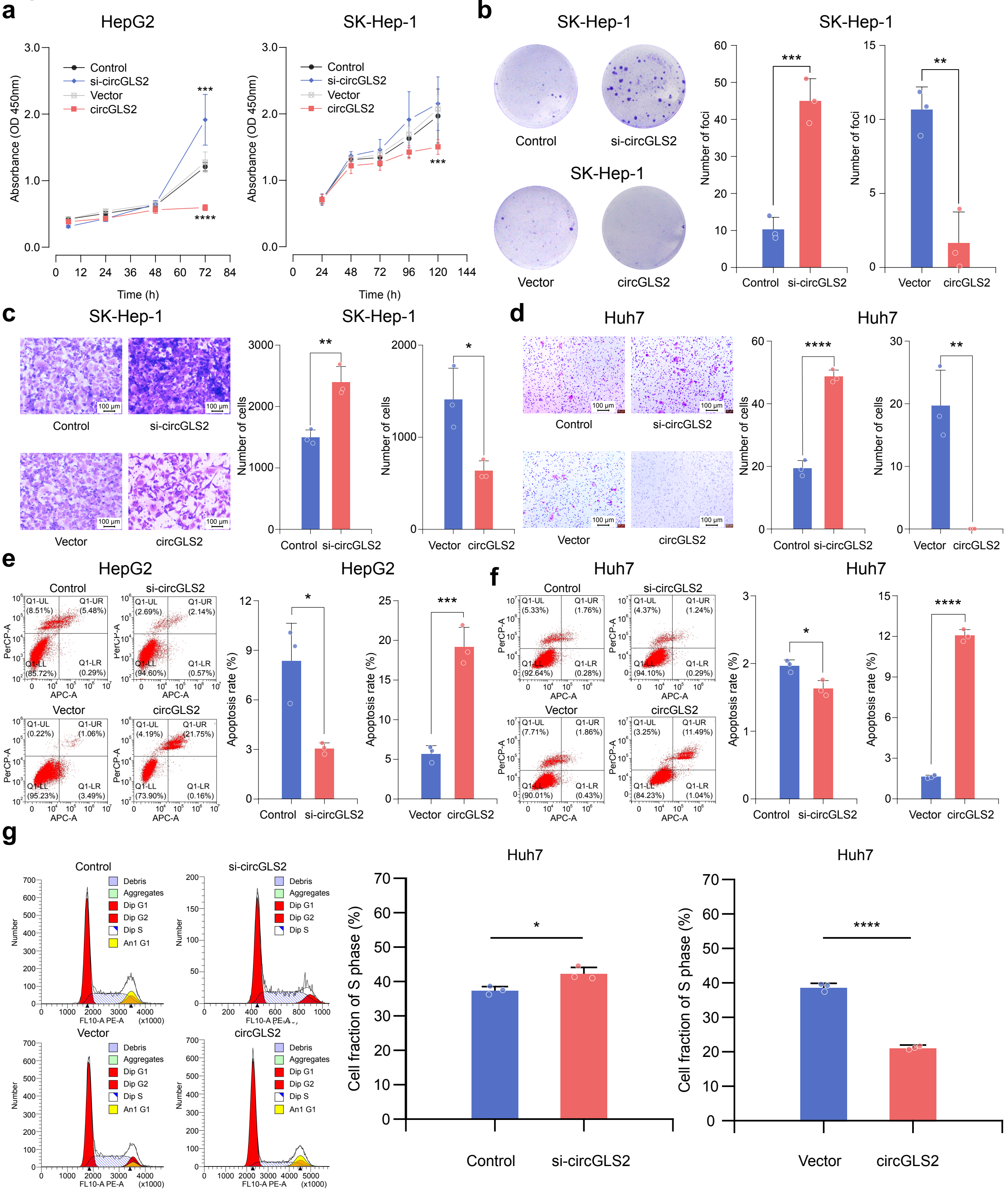

Figure. S6. Tumor suppressive characteristics of circGLS2 *in vitro*. a Cell proliferation ability of HepG2 and SK-Hep-1 with circGLS2 overexpressed or knocked down. **b** Colony formation ability of SK-Hep-1 cells with circGLS2 overexpressed or knocked down. **c**, **d** Cell migration ability of SK-Hep-1 and Huh7 cells transfected with siRNAs or overexpression plasmid of circGLS2. **e, f** Apoptosis rate alteration in HepG2 and Huh7 cells transfected with

siRNAs or overexpression plasmid of circGLS2. **g** The result of cell cycle assay in the Huh7 with circGLS2 overexpressed or knocked down. The “si-circGLS2” was the mixture of siRNAs. *: *P* value < 0.05; **: *P* value < 0.01; ***: *P* value < 1×10^{-3}; ****: *P* value < 1×10^{-4}.

Figure. S7.

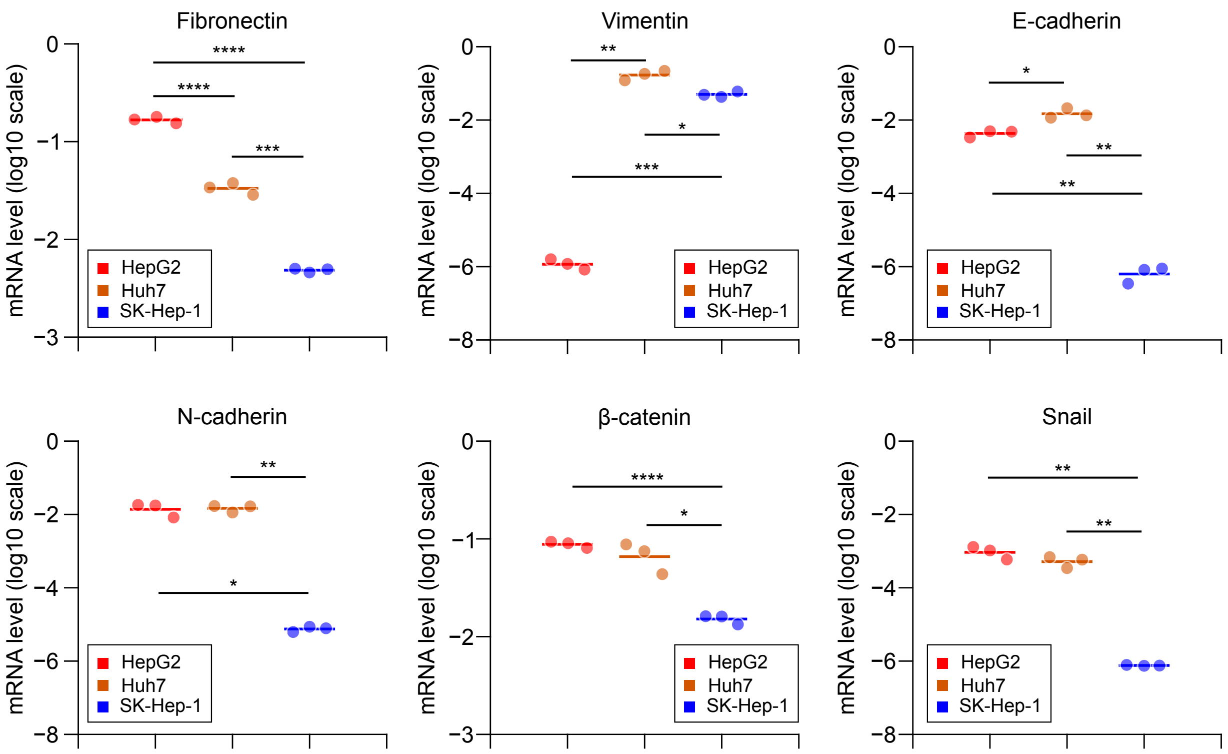

Figure. S7. The endogenous expression levels of EMT markers in three HCC cell lines. The expression levels were adjusted by those of GAPDH. *: P value < 0.05; **: P value < 0.01; ***: P value < 1×10^{-3}; ****: P value < 1×10^{-4}.

Figure. S8.

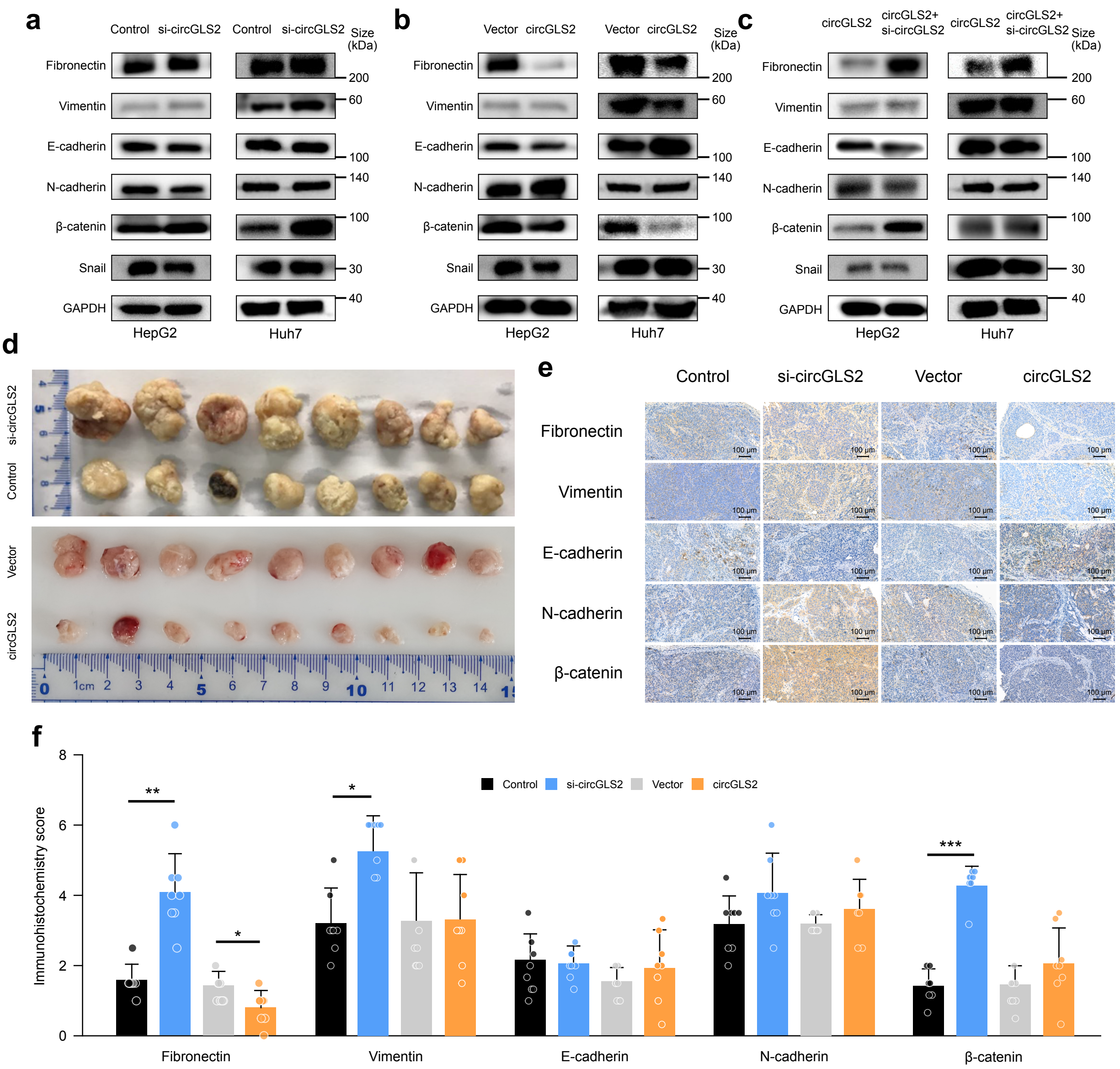

Figure. S8. circGLS2 partially attenuated epithelial–mesenchymal transition (EMT) and its tumor suppressive characteristics *in vivo*. a, b, c The abundance levels of six epithelial–mesenchymal transition markers estimated by Western blot assay in HepG2 and Huh7 transfected with siRNAs or overexpression plasmid of circGLS2. **d** The weight and volume changes of tumors collected from mice injected with circGLS2 siRNA- and overexpression plasmid-transfected Huh7. **e, f** *In vivo* EMT markers in tumor tissues stained by immunohistochemistry. The “si-circGLS2” was the mixture of siRNAs; *: P value < 0.05; **: P value < 0.01; ***: P value < 1×10^{-3}.

Figure. S9.

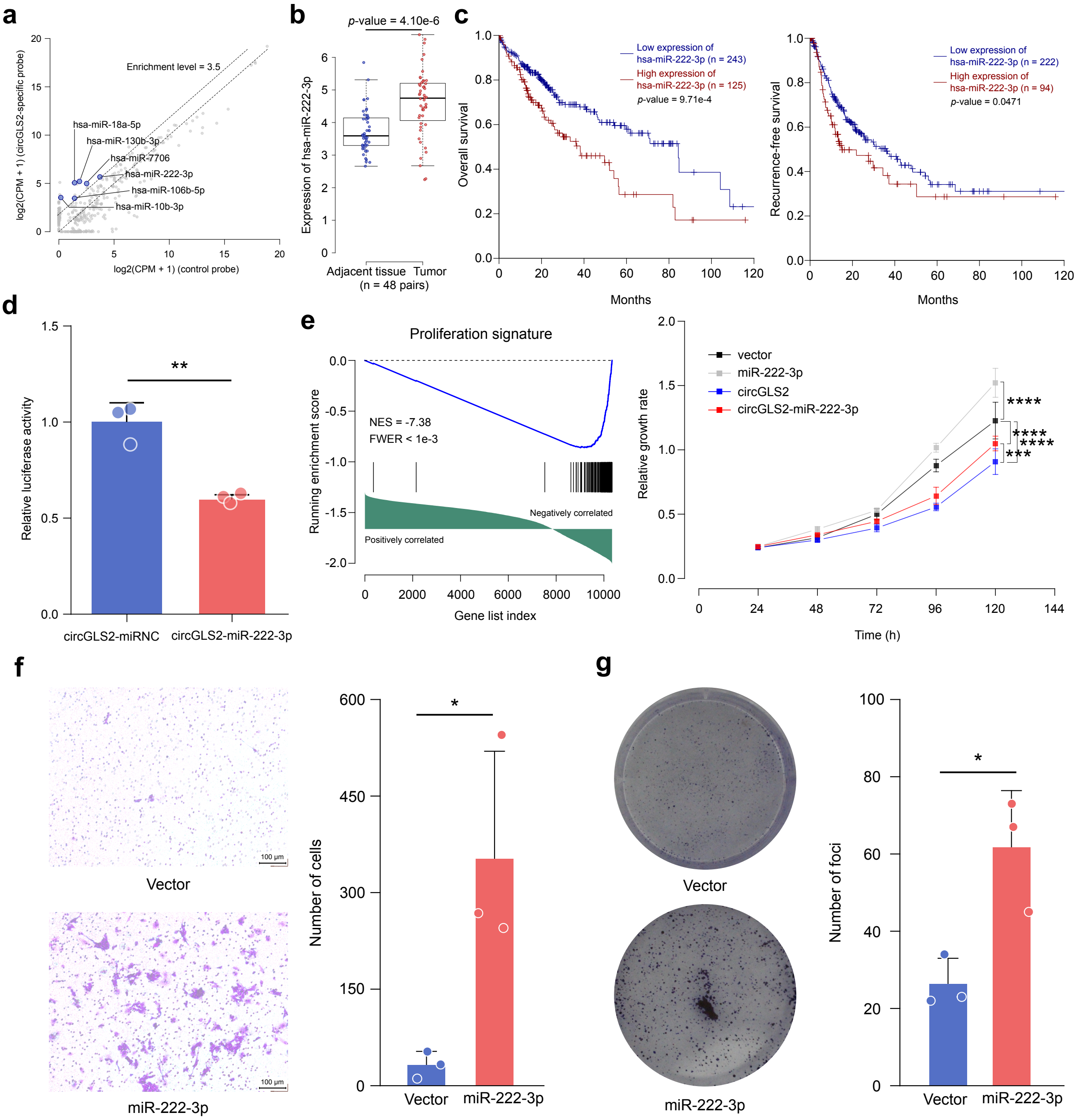

Figure. S9. circGLS2 is a sponge for hsa-miR-222-3p. a Six circGLS2-binding miRNAs obtained by RNA pulldown assay. **b** Box plot of hsa-miR-222-3p expression levels in 48 tumors and paired adjacent tissues of TCGA database. **c** (Left and Right) Kaplan–Meier curves of overall survival and recurrence-free survival for two groups of HCC patients with low and high hsa-miR-222-3p expression levels. Data visualized in Kaplan–Meier curves were retrieved from TCGA datasets. **d** Luciferase reporter assay was performed, and the relative luciferase activity was significantly decreased in the 293T cells co-transfected with overexpression plasmids of circGLS2 and hsa-miR-222-3p. **e** (Left) Enrichment plot of proliferation signature enriched by hsa-miR-222-3p. (Right) Proliferation ability of the HepG2 cells overexpressing hsa-miR-222-3p,

circGLS2, or both. **f** and **g** miR-222-3p promoted the cell migration (f) and colony formation (g) ability of HepG2 cells overexpressing miR-222-3p. *: *P* value < 0.05; **: *P* value < 0.01; ***: *P* value $< 1 \times 10^{-3}$; ****: *P* value $< 1 \times 10^{-4}$.

Figure. S10.

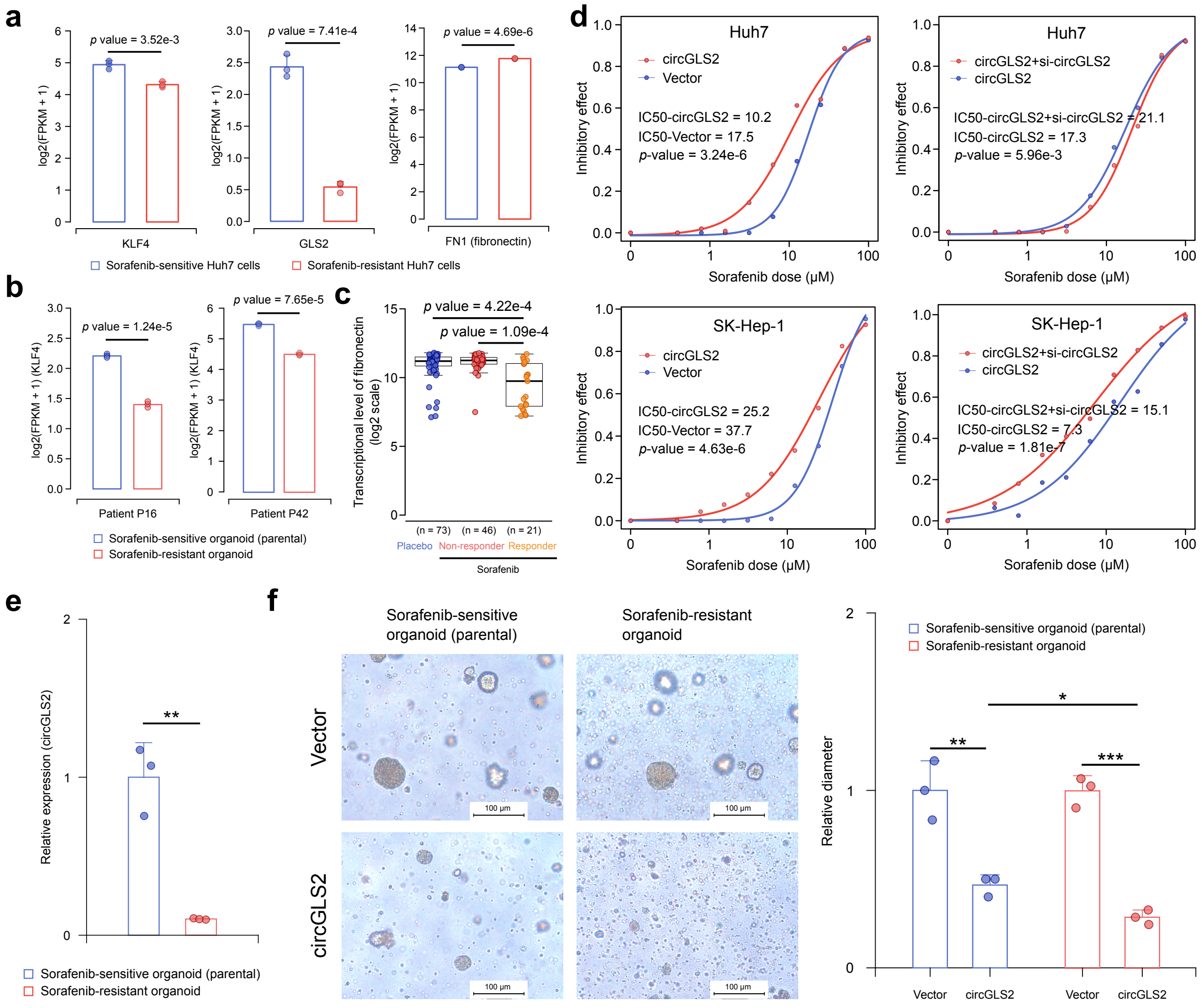

Figure. S10. circGLS2 enhanced sorafenib sensitivity of tumor cells. a The expression changes of KLF4, GLS2, and FN1 between sorafenib-sensitive and –resistant Huh7 cells. **b** The expression changes of KLF4 in two of our HCC organoids. **c** The expression change of fibronectin in the BIOSTORM microarray data. **d** The IC_{50} assay for sorafenib upon circGLS2 knockdown or overexpression in Huh7 and SK-Hep-1 cells. **e** circGLS2 level was significantly lower in the sorafenib-resistant organoid. **f** circGLS2 was overexpressed in the parental and sorafenib-resistant organoid and their growing ability was compared with each other. The “si-circGLS2” was the mixture of siRNAs. *: P value < 0.05; **: P value < 0.01; ***: P value < 1×10^{-3}.
